# Rapid turnover of pathogen-blocking *Wolbachia* and their incompatibility loci

**DOI:** 10.1101/2023.12.04.569981

**Published:** 2023-12-05

**Authors:** J. Dylan Shropshire, William R. Conner, Dan Vanderpool, Ary A. Hoffmann, Michael Turelli, Brandon S. Cooper

**Affiliations:** 1 Division of Biological Sciences, University of Montana, Missoula, Montana, USA; 2 Department of Biological Sciences, Lehigh University, Bethlehem, Pennsylvania, USA; 3 Forest Service, National Genomics Center for Wildlife and Fish Conservation, Missoula, Montana, USA; 4 Pest and Environmental Adaptation Research Group, Bio21 Institute and the School of BioSciences, The University of Melbourne, Parkville, Australia; 5 Department of Evolution and Ecology, University of California, Davis, California, USA

**Keywords:** cytoplasmic incompatibility, *Drosophila*, endosymbiosis, horizontal transmission, host switching, insertion sequence elements

## Abstract

At least half of all insect species carry maternally inherited *Wolbachia* alphaproteobacteria, making *Wolbachia* the most common endosymbionts in nature. *Wolbachia* spread to high frequencies is often due to cytoplasmic incompatibility (CI), a *Wolbachia*-induced sperm modification that kills embryos without *Wolbachia*. Several CI-causing *Wolbachia* variants, including *w*Mel from *Drosophila melanogaster*, also block viruses. Establishing pathogen-blocking *w*Mel in natural *Aedes aegypti* mosquito populations has reduced dengue disease incidence, with one study reporting about 85% reduction when *w*Mel frequency is high. However, *w*Mel transinfection establishment is challenging in many environments, highlighting the importance of identifying CI-causing *Wolbachia* variants that stably persist in diverse hosts and habitats. We demonstrate that *w*Mel-like variants have naturally established in widely distributed holometabolous dipteran and hymenopteran insects that diverged approximately 350 million years ago, with *w*Mel variants spreading rapidly among these hosts over only the last 100,000 years. *Wolbachia* genomes contain prophages that encode CI-causing operons (*cifs*). These *cifs* move among *Wolbachia* genomes – with and without prophages – even more rapidly than *Wolbachia* move among insect hosts. Our results shed light on how rapid host switching and horizontal gene transfer contribute to *Wolbachia* and *cif* diversity in nature. The diverse *w*Mel variants we report here from hosts present in different climates offer many new options for broadening *Wolbachia*-based biocontrol of diseases and pests.

Maternally transmitted *Wolbachia* bacteria were first discovered in the ovaries of the mosquito *Culex pipiens* (1) and are now recognized as the most common endosymbiont of insects, occurring in about half of all species (2). *Wolbachia*’s success stems largely from its ability to affect host reproduction to favor *Wolbachia* transmission. For example, cytoplasmic incompatibility (CI) kills *Wolbachia*-free embryos fertilized by *Wolbachia*-carrying males, driving the endosymbiont to high frequencies. Model *Drosophila* systems have facilitated understanding of *Wolbachia* population biology and CI. The first two *Wolbachia* variants discovered in *Drosophila* species were *w*Ri, found in southern California *D. simulans* (3) and *w*Mel, found in Australian *D. melanogaster* (4). Both variants rapidly spread worldwide within these invasive hosts (5–7). *w*Ri now occurs at relatively stable frequencies (~93%) in almost all characterized *D. simulans* populations (8), while the frequencies of *w*Mel and closely related variants vary significantly among host populations (9–11).

CI enables the suppression of vector and pest populations and the transformation of natural vector populations with pathogen-blocking *Wolbachia*. When *w*Mel *Wolbachia* are moved from *D. melanogaster* to *Aedes aegypti*–species diverged 250 million years ago (MYA)–CI associated with *w*Mel can drive the endosymbiont to high frequencies where it can alleviate human disease burden (12), with one study reporting an 86% reduction in dengue (13). While artificial *w*Mel transfer between divergent Diptera has led to successful disease control (13), not all attempted *w*Mel introductions have succeeded (14, 15). This raises the issue of whether alternative *w*Mel variants might spread in these situations, but the diversity of *w*Mel variants and the timescale of their host-switching remain uncertain. By comparing phylograms and chronograms for host and *Wolbachia* genomes, we document that variants closely related to *w*Mel have naturally transfered among dipteran and hymenopteran hosts 100 million years (MY) more diverged than donor *D. melanogaster* and recipient *Ae. aegypti*. These host invasions are accompanied by even faster turnover of CI-causing operons (*cifs*) via both phage-dependent and phage-independent transfer among *Wolbachia* genomes. Our results offer insights for further broadening applications of *w*Mel variants and their diverse *cifs*.

## Rapid spread of *w*Mel-like *Wolbachia* Across Hosts Diverged 350 MYA

To illustrate the timescales of *Wolbachia* movement across host species and of *cifs* among *Wolbachia* genomes, we focus on *Wolbachia* closely related to *w*Mel. We use “*w*Mel-like” to designate the clade examined here (16), comparing the results to “*w*Ri-like” *Wolbachia* (17) (Table S1). Although the clade boundaries are arbitrary, our conclusions concerning rapid movement of closely related *Wolbachia* among distantly related hosts—and rapid turnover of *Wovirus* and *cifs* within those *Wolbachia* genomes—rest on robustly estimated chronograms. When pervasive recombination and horizontal exchange were initially described among diverse *Wolbachia* (18, 19), it was conjectured that recombination precluded reliable bifurcating phylogenies and chronograms for these mosaic genomes. However, subsequent analyses cf.(20) show that despite rapid and frequent movements of CI-determining loci, phages, and other genetic elements, large portions of the *w*Mel-like and *w*Ri-like genomes show no significant evidence of recombination (Supplementary Discussion). We use these apparently quasi-clonal genomic regions for our phylogenetic and divergence-time analyses and show that previous analyses which did not explicitly control for recombination are robust (Fig. S1; Supplementary Discussion) (17, 21).

**Fig. 1.**
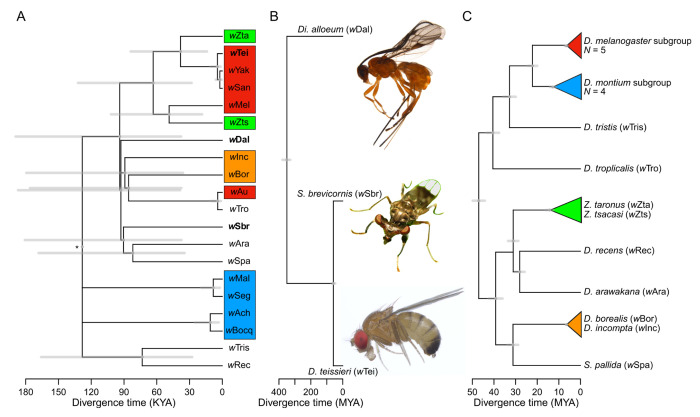
*w*Mel-like *Wolbachia* that diverged approximately 94 KYA occupy insects that diverged about 350 MYA. **(A)** An absolute chronogram with the *Wolbachia* associated with the most distantly related hosts in bold. The colored *Wolbachia* labels match the host clades from Panel C. Horizontal gray bars are 95% credible intervals of divergence times. The crown age is 128 KY, with 95% credible interval of 263 to 51 KY*. **(B)** An approximate chronogram for the most distantly related host clades containing *w*Mel-like *Wolbachia*: Hymenoptera (*Diachasma alloeum*) and within Diptera, Diopsidea (*Spyracephala brevicornis*) and Drosophilidae (*D. teissieri* presented as a representative drosophilid). Diptera and Hymenoptera diverged about 350 MYA (378–329 MYA, Devonian–Carboniferous) and span Holometabola (Misof et al. 2014; Johnson et al. 2018). Drosophilidae and the Diopsoidea superfamily containing Diopsidea stalk-eyed flies diverged about 59 MYA based on the crown age of the Drosophilidae (47 MY) (24), and the crown age of Schizophora (70 MY) (66). **(C)** A chronogram for drosophilid hosts with node ages and approximate confidence intervals estimated from the fossil-calibrated chronogram of Suvorov et al. (2022) (24). Images taken by Centre for Biodiversity Genomics (*D. alloeum*), Katja Schulz (*S. brevicornis)* and Tim Wheeler (*D. teissieri*).

We consider 20 *w*Mel-like *Wolbachia* whose host species include dipteran and hymenopteran hosts, with a most recent common ancestor (MRCA) about 350 MYA (Fig. 1A–C) (22). Using all single-copy genes of equal length with little evidence of past recombination (Supplementary Discussion), the bulk of these *Wolbachia* genomes diverged only about 128 KYA (95% credible interval: 51 – 263 KYA) (Fig. 1A). Fig. 1B shows an approximate chronogram for the most diverged hosts: a wasp, *Diachasma alloeum*; a stalk-eyed fly, *Sphyracephala brevicornis*; and *D. teissieri* as a representative drosophilid. (The divergence time of the insect orders Diptera, which includes the families Drosophilidae and Diopoidea (stalk-eyed flies), and Hymenoptera is comparable to the crown age of all extant tetrapods, ~373 MY (23). In contrast, the *w*Mel-like *Wolbachia* in these most-diverged hosts, denoted *w*Dal, *w*Sbr and *w*Mel, respectively, diverged only about 94 KYA (95% CI: 38 – 189 KYA) (Fig. 1A). We also report *w*Mel-like *Wolbachia* in 18 drosophilids (Fig. 1C), including a variant, *w*Zts, in tropical *Zaprionus tscasi* that is now the closest known relative of *w*Mel in *D. melanogaster. Zaprionus* flies are members of the *Drosophila* subgenus that diverged from the *Sophophora* subgenus about 47 MYA (95% CI: 43.9 – 49.9 MYA), highlighting *w*Mel proliferation across species that span the entire paraphyletic genus also named *Drosophila* (24), similar to *w*Ri (17). We discuss additional inferences that can be made when more than one *Wolbachia* sequence from these hosts and others become available and provide a correction of prior claims concerning the *Wolbachia* in *D. suzukii* and *D. subpulchrella* based on species misidentification (Fig. S2, Supplementary Discussion). The complete set of hosts and their *w*Mel-like *Wolbachia* are provided in Table S1.

## Rapid Introgressive Transfer of *Wolbachia* Between Some Closely Related Species

Many obligate mutualistic endosymbionts like the *Wolbachia* in filarial nematodes (25) and *Buchnera* in aphids (26) are acquired cladogenically. In contrast, all but one of these *w*Mel-like *Wolbachia* must have been acquired through introgression or non-sexual horizontal transmission. Introgression is plausible only between the most closely related drosophilid species (indicated by the colored triangles in Fig. 1C). Joint analysis of mtDNA and *Wolbachia* sequences implies that the three-species *yakuba* clade (*D. teissieri, (D. yakuba, D. santomea*)) first acquired *Wolbachia* by horizontal transmission from an unknown host, then transferred it within the clade through hybridization and introgression (27). *D. yakuba* hybridizes with its endemic sister species *D. santomea* on the island of São Tomé (10, 28, 29), and with *D. teissieri* on the edges of forests on the nearby island of Bioko (30). *Wolbachia* and mtDNA chronograms are generally concordant for these three hosts and indicate more recent common ancestors than for the bulk of the host nuclear genomes (27). *Z. taronus* co-occurs on São Tomé, particularly with *D. santomea* at high altitudes, but diverged from the *D. yakuba* triad about 47 MYA (Fig. 1C), making introgression impossible. Yet, its *Wolbachia* (*w*Zta) diverged from the *w*Yak-clade *Wolbachia* only about 14–84 KY. These data illustrate horizontal *Wolbachia* transfer between distantly related species with overlapping ranges and habitats.

Other possible examples of introgressive *Wolbachia* acquisition involve two species pairs in the *D. montium* subgroup, (*D. seguyi, D. malagassya*) and (*D. bocqueti, D*. sp. aff. *chauvacae*), whose *Wolbachia* diverged on the order of 10 KY. Both host pairs appear as sister species (31), so introgressive *Wolbachia* transfer is plausible. However, the mtDNA third-position coding sites differ by 0.53% and 1.15% respectively, corresponding to divergence times on the order of 100 KY or longer (32), inconsistent with introgressive acquisition of the corresponding *Wolbachia*. For the more distantly related pairs (*Z. taronus, Z. tsacasi*) and (*D. borealis, D. incompta*), the mtDNA third-site differences of 19.3% and 30% respectively, decisively preclude introgressive *Wolbachia* transfer.

## *w*Mel-like *Wolbachia* Hosts are Diverse and Cytoplasmic Incompatibility is Common

Notably, the hosts of these *w*Mel-like *Wolbachia* are extraordinarily diverse in terms of ecology and geography. They range from cosmopolitan human-associated species (*D. melanogaster, D. simulans* and *S. pallida*) to endemics restricted to small oceanic islands (*D. santomea* and *D. arawakana*). The drosophilids include one that breeds and feeds on flowers (*D. incompta*), a mushroom specialist (*D. recens*), and classic generalists (e.g., *D. melanogaster* and *D. simulans*). As expected, hosts with closely related *Wolbachia* co-occur somewhere – or did in the recent past. For instance, *w*Au was previously observed in *D. simulans* in Florida and Ecuador (6). Thus, before *w*Au was displaced by *w*Ri, *w*Au-carrying *D. simulans* probably co-occurred with *D. tropicalis* found only in Central and South America and Caribbean islands, which harbors *w*Tro, sister to *w*Au in Fig.1C. Although the wasp *Diachasma alloeum* parasitizes the tephritid *Rhagoletis pomonella*, none of *R. pomonella*’s several *Wolbachia* seem to be *w*Mel-like (33).

**Fig. 2.**
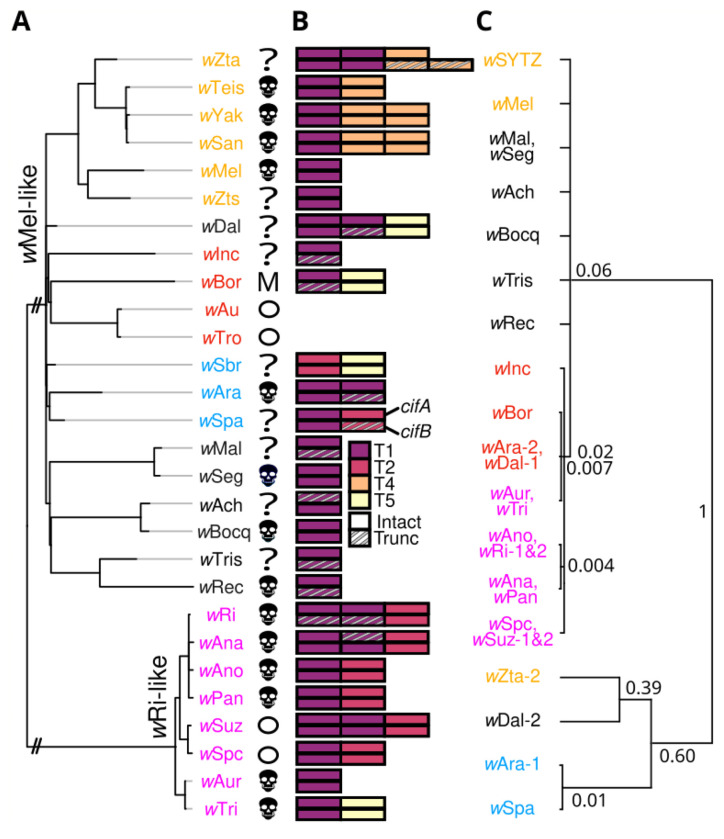
Diverse *cif* operons rapidly turnover among *w*Mel-and *w*Ri-like genomes. **(A)** A phylogram of *w*Mel-like and *w*Ri-like *Wolbachia*, including variants that cause CI (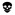), do not cause CI (circles), or have unknown CI status (?). *w*Bor does not cause CI, but it does kill males (M). The *w*Mel-like and *w*Ri-like clades diverged about 1. 1 MYA (see SI). Branches leading to these clades are shortened (//) and light gray branch extensions are used to improve visualization. **(B)** These closely related Wolbachia carry four of five known *cif* operon Types (T1-T5). *cifA* (top) and *cifB* (bottom) schematics are presented with operon copies adjacent to one another. **(C)** A relative chronogram for *cifA_[T1]_* with node labels indicating relative ages, scaled to 1 for the most diverged. Identical sequences are collapsed into a single tip, and nodes with posterior probability < 0.95 are collapsed into polytomies. Strain labels are colored to highlight *Wolbachia-cifA* discordance.

We analyzed the phylogenetic distribution of CI—and the *cif* genes underlying it (34–37). CI contributes to *Wolbachia* incidence among and frequency within species (38), as well as *Wolbachia* persistence within both natural and transinfected hosts (13, 39, 40). Of our 20 *w*Mel- and 8 *w*Ri-like *Wolbachia*, all but 11 *w*Mel-like strains have been tested for CI. For 2 of these 11 *w*Mel-like strains (*w*Seg in *D. seguyi* and *w*Bocq in *D. bocqueti*), stocks were available for us to test for CI. Comparing hatch rates of embryos produced by the CI cross to hatch rates of embryos from the three compatible crosses, we found that *w*Seg and *w*Bocq cause relatively strong CI, with relative hatch rates from incompatible crosses less than half that from compatible crosses. Including these two new CI systems, 8 *w*Mel-like and 6 *w*Ri-like *Wolbachia* in our study cause CI (Fig. 2A; see Table S1 for references). CI strength varies greatly among these systems and others (41), but may also vary within systems as a function of male age (42), environmental factors (34), and host backgrounds (10, 34, 42, 43). As an example, *w*Mel causes strong CI in *D. simulans* (44) and *Ae. aegypti* (12) backgrounds, but generally weak CI in its source host, highlighting *w*Mel’s ability to cause strong CI in hosts that have presumably not evolved to suppress *w*Mel-induced CI. Additional large-scale tests for CI are needed for *Wolbachia* that reportedly do not cause CI, but maintain *cifs*, as these strains may cause CI under some conditions.

## *cif* Genes and Proteins are Highly Diverse Among *w*Mel-like *Wolbachia*

CI is caused by two-gene *cif* operons, with paternal expression of *cifB* (and occasionally *cifA*) killing embryos unless a complementary *cifA* copy is expressed maternally (35–37, 45–49). *cifs* are generally associated with *Wovirus* bacteriophages that are themselves subdivided into four groups typed using serine recombinase (sr) alleles (sr1WO–sr4WO), with three harboring *cifs* (sr1WO-sr3WO) (50). *Wolbachia*-encoded *cifs* span five described clades called Types (*cif*_[T1]_–*cif*_[T5]_) (51), and individual *Wolbachia* genomes often include multiple *cif* operons (Fig. 2B) (51, 52). Excluding non-CI-inducing *w*Mel-like *w*Au and *w*Tro (6, 53), all other *Wolbachia* in our analyses encode between one and three *cif* operons from four of the five described *cif* types: all genomes except *w*Sbr contain a *cif*_[T1]_ operon, eight contain a *cif*_[T2]_ operon, and *cif*_[T4]_ and *cif*_[T5]_ operons each appear in four. *w*Mel-like and *w*Ri-like *Wolbachia* do not encode *cif*_[T3]_] operons (Fig. 2B). Even within *cif* types, there is significant variation in protein sequence, length, and domain composition (Fig. S3A–C; Supplementary Discussion). An exceptional example is CifB_*w*Zta[T1-2]_ (i.e., the second copy of CifB_[T1]_ found in *w*Zta). It is 357 amino acids longer than the next largest CifB_[T1]_ and shares only 51% to 39% sequence identity with other CifB_[T1]_ proteins (Table S2). Despite its considerable divergence, *w*Zta’s CifB is more similar to other CifB_[T1]_ proteins in our dataset than to any other putatively functional CifB Types, and retains a pair of PD-(D/E)XK nuclease domains ubiquitous among CifB proteins.

## *cif* Operons Turn Over Rapidly Among Closely Related Wolbachia

As previously noted, the phylogenies of *cifs* and the *Wolbachia* that contain them are discordant (27, 35, 51); but how rapidly do *cifs* turnover among *Wolbachia* genomes? In the *w*SYTZ clade (*w*SYT plus *w*Zta), *w*SYT genomes contain only one *cif*_[T1]_ operon but *w*Zta contains two, indicating a gain or loss in the last 14,000–84,000 years (Fig. 2). The level of divergence between *w*Zta’s two *cif*_[T1]_ operons rules out duplication as an origin of the second pair (Fig. 2C). Turnover is not restricted to particular *cif* types, as exemplified by the *w*SYTZ clade acquiring *cif*_[T4]_ operons since it diverged from (*w*Mel, *w*Zts) 28–132 KYA; moreover, in each *Wolbachia,* the *cif*_[T4]_ operons occur in the same phage as the *cif*_[T1]_ operon. *w*Yak and *w*San diverged from *w*Tei only 1.5–10 KYA, yet they have a second *cif*_[T4]_ operon (54). The trio ((*w*Ara, *w*Spa), *w*Sbr), which diverged 37–181 KYA, also have several notable changes. *w*Ara contains two *cif*_[T1]_ operons, while its sister *w*Spa contains a *cif*_[T1]_ operon and a *cif*_[T2]_ operon. The outgroup in this trio, *w*Sbr, does not contain a *cif*_[T1]_ operon, but instead contains *cif*_[T5]_ and *cif*_[T2]_ operons. The trio ((*w*Ara, *w*Spa), *w*Sbr) illustrates turnover of *cif*_[T1]_, *cif*_[T2]_] and *cif*_[T5]_ operons within 28–132 KY. The variants (*w*Inc, (*w*Bor, (*w*Au, *w*Tro))), which diverged 36–179 KYA, indicate turnover, loss and gain of *cifs*: both *w*Inc and *w*Bor contain a *cif*_[T1]_ operon, only *w*Bor contains a *cif*_[T5]_ operon, while sister *w*Au and *w*Tro contain no *cifs*. Finally, the *w*Ri-like clade, which diverged 5–27 KYA (17), displays comparably rapid *cif* turnover. Most dramatically, the pair (*w*Aur, *w*Tri), which diverged only 13–3400 years ago (Supplementary Discussion), differ in that only *w*Tri contains a *cif*_[T5]_ operon. Overall, diverse *cif* operons move among closely related *Wolbachia* even more rapidly than these *Wolbachia* move among distantly related hosts.

We further illustrate rapid *cif* turnover by focusing on homologs of *cifA*_[T1]_. *cif*_[T1]_ variants are the most common *cifs* among our *w*Mel-like and *w*Ri-like *Wolbachia*. We estimate the *w*Mel-like and *w*Ri-like clades diverged about 450 K-1.1 MYA (SI). Among *w*Mel-like variants, 11 contain very similar *cifA*_[T1]_ on a polytomy closely related to *cifA*_[T1]_ of the *w*Ri-like variants (Fig. 2C). The *w*Ri-like clade, with MRCA 5–27 KYA, contains very similar *cifA*_[T1]_ alleles, suggesting that the *cif*_[T1]_ homologs are codiverging with these *w*Ri-like *Wolbachia*. In contrast, the *cifA*_[T1]_ alleles of the *w*Mel-like variants *w*Dal, *w*Ara, *w*Bor and *w*Inc are more similar to the *cifA*_[T1]_ of *w*Ri-like *w*Aur and *w*Tri (99.8–99.2% aa identity) than to the *cifA*_[T1]_ alleles found in the 11 *w*Mel-like *Wolbachia* in the polytomy (Fig. 2C). This suggests a recent transfer of *cif*_[T1]_ operons between the *w*Mel-like and *w*Ri-like clades, or from a third unknown source. Closely related *cifA*_[T1]_ variants are in *w*Mel-like *w*Dal, *w*Ara, *w*Spa, and *w*Zta. The grouping of *w*Zta and *w*Dal again reflects the flux of *cif*_[T1]_ loci across *w*Mel-like *Wolbachia*.

## Wovirus Turnover Does Not Fully Explain *cif* Movement

**Fig. 3.**
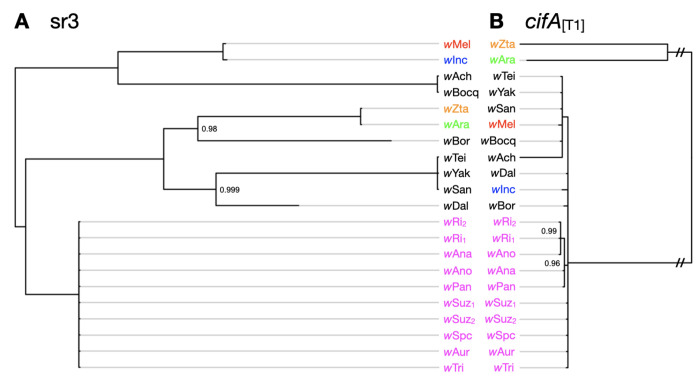
Discordant phylograms for sr3 alleles of sr3WO and linked *cifA_[T1]_* alleles demonstrate phage-independent cif turnover. **(A)** Phylogram for sr alleles facing **(B)** phylogram for *cifA_[T1]_* alleles linked to these sr3 alleles. Branches leading to the two sets of closely related *cifA_[T1]_* alleles are shortened (//) to improve visualization. *w*Ri-like strains are shown in magenta, and focal *w*Mel-like strains are colored to highlight sr3-*cifA_[T1]_* discordance. Subscripts represent different sr3 copies within the same *Wolbachia* genome (see Table S3) in cases where multiple sr3 alleles can be associated with specific *cifA_[T1]_* copies. Subscripts presented for *cifA_[T1]_* alleles denote associated sr alleles. Light gray branch extensions are provided to simplify sr3-*cifA_[T1]_* comparisons. Nodes with posterior probability < 0.95 are collapsed into polytomies. Posterior support values appear only at nodes with support less than 1.

Two additional aspects of our data on *cif* transfer are worth emphasizing. First, the *Wovirus* bacteriophages that contain *cifs* turnover among *Wolbachia* genomes (Supplementary Discussion) on the order of 1–100 KY. An exceptional case involves the *w*Bocq genome that contains an sr3WO *Wovirus* that is absent from the genome of sister *w*Ach. This implies gain or loss of this *Wovirus* by (*w*Bocq, *w*Ach) in the last 3.3–25 KYA. Second, while *cifs* clearly transfer along with the *Wovirus* that carry them, *cifs* also move among divergent *Wovirus* classes (i.e., phage-independent *cif* turnover) (27)cf.(54). This interchange is documented in two ways: closely related *cifs* are found in distantly related phages and distantly related *cifs* are found in closely related phages (Fig. 3, and Supplementary Discussion). We demonstrate this by comparing phylograms of the serine recombinase genes (sr) of sr3WO *Wovirus* (sr3) to phylograms of *cifA*_[T1]_ alleles associated with them. The ten sr3WO *Wovirus* in *w*Ri-like *Wolbachia* have identical sr3 alleles and are closely related to sr3 alleles found in several *w*Mel-like *Wolbachia*. These sr3 alleles are more distantly related to several other sr3 alleles that include a copy found in *w*Mel from *D. melanogaster*. In contrast, almost all *cifA*_[T1]_ alleles associated with *w*Mel-like sr3WO *Wovirus* are very closely related to *cifA*_[T1]_ alleles associated with *w*Ri-like sr3WO *Wovirus* (Fig. 3). This generalizes phage-independent *cif* transfer among divergent *Wolbachia* as first documented for Type IV loci in *w*Yak by Cooper et al. (2019) (27) and later misinterpreted by Baião et al. (2021) (54) (Supplementary Discussion). Higher quality assemblies for known donor and recipient *Wolbachia* will be essential for establishing the relative role of insertion sequence (IS) elements (27) and other factors like plasmids in this transfer. We hypothesize a critical role for IS elements in the phage-independent *cif* transfer we document, as supported by IS elements flanking the majority of *cifs* in our analyses (Table S3).

## Selection Acts to Preserve *cifA* and Nuclease Domains within *cifB*

What is the fate of these *cifs*? Theory predicts that once *Wolbachia* are established in a host species, natural selection does not act to maintain CI but does act to maintain resistance to CI (55, 56). Consistent with these predictions and prior observations (21, 51), Fig. 2B shows that putative pseudogenization (i.e., truncation) is more common for *cifB* than for *cifA* (see also Fig. S4A,B). Still, as noted by Beckmann et al. (2021) (57), CI is incredibly common despite weak selection on the phenotype (34). This paradoxical prevalence of CI across *Wolbachia* lineages can be explained in part by clade selection in which CI-causing *Wolbachia* lineages are more likely to be transmitted to new host species because they typically have higher frequencies within host species and persist longer than do non-CI causing *Wolbachia (38).* However, CifB also contributes to alternative functions that include *Wolbachia* titer regulation through interactions with host autophagy (58). This suggests that Cif moonlighting could plausibly contribute to preservation of particular *cifs*.

**Fig. 4.**
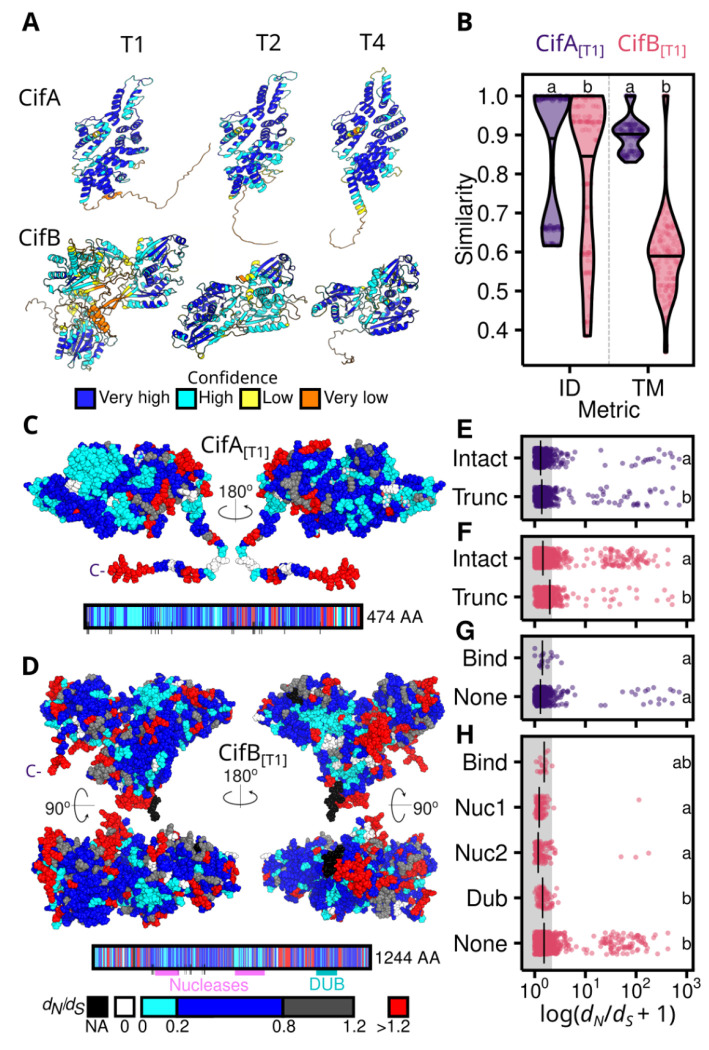
Cifs are highly variable and CifA tends to have lower values of *d_N_/d_S_* than CifB. **(A)** Representative Cif_*w*Mel[T1]_, Cif_*w*Ri[T2]_, and Cif_*w*Yak[T4]_ AlphaFold structures colored by confidence (pLDDT) per residue. **(B)** Putatively intact CifA_[T1]_ proteins are more similar than CifB_[T1]_ from the same pairs (*N* = 18) in terms of both sequence identity (ID) and structural similarity (TM). *d_N_*/*d_S_* for **(C)** CifA_[T1]_ and **(D)** CifB_[T1]_ displayed on Cif_*w*Mel[T1]_ AlphaFold structures and linear schematics. Median *d_N_*/*d_S_* per residue is calculated using a 10 Å spherical sliding window in pairwise comparisons of Cif_*w*Mel[T1]_ to other Cif_[T1]_ proteins (CifA: *N* = 9; CifB: *N* = 4). Colored boxes and black lines below schematics indicate domains and CifA-CifB binding sites, respectively. *d_N_*/*d_S_* is relatively lower for intact than truncated **(E)** CifA_[T1]_ and **(F)** CifB_[T1]_. **(G)** *d_N_*/*d_S_* for CifA_[T1]_ binding sites tends to not differ from *d_N_*/*d_S_* for other residues. **(H)** *d_N_*/*d_S_* tends to be lower for CifB_[T1]_ nuclease domains than for other residues. Shared letters within plots b and e-h represent statistically similar groups determined by a Mann-Whitney U test (2 groups) or a Kruskal-Wallis and Dunn’s multiple comparison test (>2 groups), *P*-values are presented in Table S4.

To assess patterns of selection across Cifs, we calculated the ratio of non-synonymous (*d_N_*) to synonymous substitutions (*d_S_*) for each Cif protein, using a 3-dimensional spherical sliding window across the length of AlphaFold-derived Cif structures (Fig. 4A, Fig. S3, Fig. S4C,D, Movie 1). Fig. 4B shows that CifA_[T1]_ proteins are more similar to one another in terms of both sequence identity and structural similarity than CifB_[T1]_ proteins from the same pairs. As predicted, CifA of Types 1 and 2 had lower *d_N_*/*d_S_* ratios than did CifB of the same type (Fig. 4, S3D–E) (Supplementary Discussion), consistent with purifying selection maintaining CifA. Putatively pseudogenized CifA and CifB proteins have higher *d_N_*/*d_S_* ratios than do intact proteins (e.g., CifA *d_N_*/*d_S_*~ 1; Fig. 4E–F), further supporting the presumption that in-frame stop codons interfere with Cif function (Supplementary Discussion). In contrast, Types 3 and 4 CifA and CifB have comparable *d_N_*/*d_S_* ratios within each type, which could plausibly stem from pleiotropy or loss-of-function (Fig. S3D–E). CifA_[T1]_ and CifB_[T1]_ binding residues and CifB_[T1]_]’s Deubiquitinase domain have *d_N_*/*d_S_* ratios comparable to non-domain associated residues. However, the two CifB_[T1]_ nuclease domains both have lower *d_N_*/*d_S_* ratios than do other residues (Fig. 4C–D, G–H). Indeed, across Cif Types, CifB’s first nuclease domain has lower *d_N_*/*d_S_* ratios than non-domain associated residues (Fig. S3F–H). While CifB’s nuclease activity may not always contribute to observed CI expression (59), selection may still act to maintain other nuclease-associated features (e.g., DNA binding) and contribute to CifB’s association with chromatin restructuring (Supplementary Discussion) (60, 61). Interestingly, our data reveal that sites with *d_N_*/*d_S_* ratios above 1, consistent with positive selection, are primarily located within regions of the protein that are not domain-associated (Fig. 4D). Many of these sites appear at the surface, aligning with the expectation that surface residues are key to host-microbe interactions, potentially facilitating interactions between Cif and host proteins. Thus, while theory predicts that selection does not act on the CI phenotype (55), selection on alternative CifB functions may plausibly delay mutational disruption of *cifB* (57).

## Conclusions

Our findings confirm that non-sexual horizontal *Wolbachia* acquisition—and introgressive transfer between close relatives—commonly occurs on the order of 10–100 KY. These conclusions are robust to uncertainties about *Wolbachia* divergence times. Among recently diverged *Wolbachia*, distantly related *cif* operons rapidly turnover via phage-mediated and phage-independent transfer. The commonness of non-sexually acquired *Wolbachia* and *cif* transfer among *Wolbachia* genomes indicates that opportunities for horizontal transfer must be common. Because many insects carry vertically transmitted *Wolbachia* (2), we expect that ephemeral somatic infections are common cf.(62). Future analyses should focus on understanding the ecology of non-sexual *Wolbachia* transfer and the cellular-genetic basis of successful non-sexual *Wolbachia* establishment (or not) in new hosts, as well as on *Wovirus/cif* transfer between *Wolbachia* genomes.

*Wolbachia* offer a practical mechanism for mitigating human diseases. While *w*Mel introductions into *Ae. aegypti* have been very effective where they have spread to high frequencies (13, 39), alternative *Wolbachia*-based interventions are needed. For example, wMel has been lost in some release locations (14, 15), particularly under extremely hot conditions. Temperature can affect CI strength (43, 63), rates of imperfect *Wolbachia* transmission (11, 64), and *Wolbachia* effects on host behaviors (16). Identifying strong-CI-causing and virus-blocking *Wolbachia* from the tropics could facilitate *Wolbachia* biocontrol, and *w*Mel-like variants that naturally associate with tropical host species are obvious candidates (49, 65). Our study introduces a comprehensive panel of closely related *w*Mel-like *Wolbachia* that exhibit diverse ecology, geography and *cif* profiles. The discovery of a tropical *Wolbachia* variant (*w*Zts), sister to *w*Mel in *D. melanogaster,* may be immediately useful for applications. While *w*Zts CI has not yet been tested, all six *Z. tscasi* sampled in nature carry *w*Zts (producing 0.61 as the 95% lower bound for *w*Zts frequency), consistent with strong CI. This versatility contributes to moving towards more customized and environment-specific *Wolbachia* applications.

## Supplementary Material

Supplement 1

Supplement 2

Supplement 3

Supplement 4

Supplement 5

Supplement 6

## Data Availability

Source data for the main and Supplementary Data figures are provided in the online version of this paper or in Dryad. Newly sequenced *Wolbachia* genomes are available under BioProject PRJNA1021588.

## Significance Statement

*Wolbachia* bacteria transinfected into *Aedes aegypti* mosquitoes (in particular the *w*Mel *Wolbachia* strain) are being widely released to suppress the transmission of dengue and other insect-vectored arboviruses. The bacterial strain *w*Mel originated from *Drosophila melanogaster,* but very little is known about its distribution and spread to other drosophilids and distantly related insects. Here we show that there has been a rapid rate of spread of *Wolbachia* strains closely related to *w*Mel not just into other flies but also into wasps, indicating a large pool of *w*Mel variants that can be employed in vector-borne disease control. *Wolbachia* prophage genes essential to this spread have evolved particularly rapidly.
