# Supplementary material for: Rapid turnover of pathogen-blocking Wolbachia and their incompatibility loci
Source: bioRxiv. 2023 Dec 5:2023.12.04.569981. Preprint. [Version 1] doi: 10.1101/2023.12.04.569981 (PMC10723362; doi:10.1101/2023.12.04.569981)
Supplement: Supplementary file 1 [file media-1.pdf]

Materials and Methods

Wolbachia assembly and phylogeny estimation

The *Wolbachia* genomes not novel to this study were obtained from the sources listed in Table S1. For the genomes novel to this study or where the source accession is an SRR number (raw reads from NCBI's SRA database), we trimmed the reads with Sickle v. 1.3 (*1*) and assembled them with ABySS v. 2.2.3 (*2*) with Kmer values of 51, 61, ..., 91. From these host assemblies, scaffolds with best nucleotide BLAST matches to known *Wolbachia* sequences, with E-values less than 10^{-10}, were extracted as the *Wolbachia* assembly. For each host, the best *Wolbachia* assembly (fewest scaffolds and highest N50) was kept. To assess the quality of these draft assemblies, we used BUSCO v. 3.0.0 to search for orthologs of the near-universal, single-copy genes in the BUSCO proteobacteria database. As a control, we performed the same search using the reference genomes for *w*Ri, *w*Au, *w*Mel, *w*Ha and *w*No.

To identify genes for the phylogenetic analyses, all of the *w*Mel-like *Wolbachia* genomes (see Table S1) were annotated with Prokka 1.14.5 (*3*), which identifies orthologs to known bacterial genes. To avoid pseudogenes, paralogs and frameshifts, we used only genes present in a single copy in each genome and required that at most one draft genome had gaps in the alignment. Genes were identified as single copy if they uniquely matched a bacterial reference gene identified by Prokka and aligned with MAFFT v. 7 (*4*). 438 genes, a total of 409,848 bp, met these criteria. These genes also show little evidence for recombination (see below).

Wolbachia chronograms

To estimate the absolute chronogram for *w*Mel-like *Wolbachia* (Fig. 1A), we first estimated a relaxed-clock relative chronogram with RevBayes 1.1.1 (*5*) with the root age fixed to 1 using the GTR + Γ model, partitioned by codon position, using the same birth-death prior as Turelli et al. (2018) (*6*). There were not enough substitutions to partition by gene as well as codon position. Each partition had an independent rate multiplier with prior $\Gamma(1,1)$, as well as stationary frequencies and exchangeability rates drawn from flat, symmetrical Dirichlet distributions. For each branch, the branch-rate prior was $\Gamma(7,7)$, normalized to a mean of 1 across all branches. Four independent runs were performed, which always agreed. Nodes with posterior probability less than 0.95 were collapsed into polytomies. We converted this relative chronogram into an absolute chronogram using the scaled distribution $\Gamma(7,7) \times 6.87 \times 10^{-9}$ substitutions per third position site per year, derived from the posterior distribution estimated by Richardson et al. (2012) (*7*), assuming 10 generations per year. Absolute branch lengths were calculated as the relative branch length times the third position rate multiplier divided by the substitutions per third-position site per year estimate above.

We produced two additional chronograms. First, we produced a chronogram using these same methods to estimate the collective divergence time of the *w*Mel-like and *w*Ri-like clades. This included all strains presented in Fig. 2. We then produced a chronogram using these same methods to estimate divergence times of the *w*Aur-*w*Tri sister pair that has not been previously estimated. This analysis included only the *w*Ri-like *Wolbachia* presented in Fig. 2.

Detecting recombination and its influence on estimated phylograms and chronograms

Homologous recombination via transformation, phage-mediated DNA exchange, and conjugation is common in bacteria (*8*–*12*), and prior work using only a few genes has identified a pattern of pervasive recombination between relatively diverged *Wolbachia* variants (*13*). To test for recombination across the *Wolbachia* genome, we used a genetic algorithm (GARD) (*14*), plus three other statistical methods implemented in PhiPack (*15*). We focused our analyses on single-copy genes that were at least 300 bp in length, with minimum recombination segments of 100 bp. We set GARD to detect a maximum of two

potential breakpoints. We first completed these analyses using our 20 *w*Mel-like variants to determine the extent of recombination between these closely related *Wolbachia*. 411 genes met our criteria for this analysis.

To test for recombination between more distantly related *Wolbachia*, we completed a second analysis using *w*Mel and *w*Ri, plus three other A-group strains (*Wolbachia* associated with *Andrena hattorfiana*, *Anoplius nigerrimus*, and *Apoderus coryli*), and B-group *w*Mau in *Drosophila mauritiana* (*16*, *17*). We searched for homologs of all 1292 genes in *w*Mel in these five other *Wolbachia*. Homologs for 1124 genes were found in all 5 *Wolbachia*, of which 111 were shorter than 300pb and excluded. The remaining 1013 were tested for recombination using GARD and the three statistical tests implemented in PhiPack.

Because recombination could potentially influence our estimation of phylograms and chronograms, we also revisited the phylogram and chronogram analyses presented in Meany et al. (2019) (*17*) that included 9 group-A and 6 group-B strains. We identified genes with no evidence of recombination according to all 4 tests described above and used them to re-estimate a Bayesian phylogram and an absolute chronogram with RevBayes 1.1.1, as in Meany et al. (2019) (*17*). We estimated the phylogram with the GTR + Γ model, partitioning by codon position (*5*). To estimate the absolute chronogram, we first estimated a relative relaxed-clock chronogram with the root age fixed to 1, partitioned by codon position. The relaxed-clock branch-rate prior was Γ(7,7), normalized to a mean of 1 across all branches. We transformed the relative chronogram into an absolute chronogram using a substitution-rate estimate of $\Gamma(7,7) \times 6.87\times10^{-9}$ substitutions/3rd position site/year. This rate estimate was chosen such that the upper and lower credible intervals matched the posterior distribution estimated by Richardson et al. (2012) (*7*), assuming 10 generations/year, normalized by their median estimate of 6.87×10^{-9} substitutions/3rd position site/year.

Host phylogeny and chronograms

A key conclusion of our analyses is that closely related *Wolbachia* move among distantly related hosts on a time scale many orders of magnitude faster than host divergence times. Our estimates concerning host divergence rely on recent calibrations from the literature. Fig. 1B presents our most diverged hosts. The clades Diptera and Hymenoptera span the Holometabola. According to the fossil-calibrated chronograms in Wang et al. (2016, their Fig. 3), Diptera and Hymenoptera diverged ~350 million years ago (MYA), with 95% highest posterior density credibility interval (HPD CI) of (378–329 MYA, Devonian–Carboniferous). This is consistent with the point estimates produced by Misof et al. (2014) (*18*) and Johnson et al. (2018) (*19*) (Fig. 1). The placement of the family Diopsidea, the stalk-eyed fly clade that includes *Sphyracephala bevicornis*, within superfamily Diopsoidea in the paraphyletic acalyptrate group of Schizophora remains uncertain (*20*). Hence, the maximum divergence time between *Sphyracephala bevicornis* and any drosophilid is the crown age of the Schizophora, which certainly includes both the Drosophilidae and Diopsoidea. The minimum divergence time between the Drosophilidae and Diopsoidea is the crown age of the Drosophilidae (which certainly excludes the Diopsoidea). Wiegmann et al. (2011) (*20*) (Fig. 3) estimate the crown age of the Schizophora at ~70 MYA. Suvorov et al. (2022) (*21*) (Fig. 1) estimate the crown age of the Drosophilidae at about 47 MYA (with 95% HPD CI of 43.9–49.9, Devonian–Carboniferous). Our approximate point estimate in Fig. 1B for the divergence of Drosophilidae and Diopsoidea, 59 MY, is the midpoint of these bounds.

For the drosophilids in Fig. 1C, node ages and approximate confidence intervals were estimated from the fossil-calibrated chronogram of Suvorov et al. (2022) (*21*), using supplementary information as needed. We number the 12 nodes in Fig. 1C from left to right, with 1 denoting the divergence between the subgenera *Sophophora* (including *D. tropicalis*) and *Drosophila* (including *D. arawakana*), 2 denoting the divergence of *D. tropicalis* from the *D. melanogaster* subgroup, …, 11 denoting the crown age of the *D. melanogaster* subgroup, and 12 denoting divergence time between *D. borealis* and *D. incompta*. For species in our Fig. 1C that are not included in Fig. 1 of Suvorov et al. (2022) (*21*), we used the NCBI

Taxonomy Browser to determine the closest relative(s) included in Suvorov et al. (2022) (*21*). We estimated node ages and approximate CIs from the x-axis of their Fig. 1, using the measurement tool in Adobe Acrobat Pro DC (ver. 2022.001.20169), converting distances to time using the scale bar at the bottom of their figure. Our symmetrical approximate CIs were obtained by measuring the widths of the CI profiles in Fig. 1 of Suvorov et al. (2022) (*21*). This method produced the approximate ages and CIs for nodes 1–11 in our Fig. 1C. As a check, our approximation method produces 47 ± 2.8 MY as the crown age in Fig. 1C; in their text, Suvorov et al. (2022) (*21*) estimate this age as ~47 MYA with 95% CI 43.9–49.9 MYA.

We used our Suvorov et al. (2022) (*21*) calibrations to set the crown ages of the *melanogaster* and *montium* subgroups (nodes 10 and 11). Within those subgroups and for node 12 (*D. borealis*, *D. incompta*), we estimated relative divergence using relaxed-clock relative chronograms under a GTR + Γ [7,7] model of molecular evolution, following the methods in Conner et al. (2021) (*22*), summarized below. Estimating the divergence time between *D. borealis* and *D. incompta* was the most problematic. *D. incompta* belongs to the *D. flavipilosa* species group (*23*). Both nuclear and mtDNA data indicate that among the host species we analyzed, *D. incompta* is most closely related to *D. borealis* in the *D. virilis* species group.

To estimate relative divergence times for host drosophilids not included in Suvorov et al. (2022) (*21*), we obtained genomes from NCBI (Table S1). Coding sequences for the 20 nuclear genes used in the analyses of Turelli et al. (2018) (*6*) (*aconitase, aldolase, bicoid, ebony, Enolase, esc, g6pdh, GlyP, GlyS, ninaE, pepck, Pgi, Pgm1, pic, ptc, Tpi, Transaldolase, white, wingless,* and *yellow*) were obtained from FlyBase for *D. melanogaster*. We used tBLASTn to identify orthologs in the other genome assemblies. The sequences were aligned with MAFFT v. 7 (*4*) and trimmed of introns using the *D. melanogaster* sequences as a guide. We estimated a relaxed-clock relative chronogram with RevBayes 1.1.1 (*5*) with the root age fixed to 1 using the GTR + Γ [7,7] model, partitioned by gene and codon position. We used the same birth-death prior as Turelli et al. (2018) (*6*). Each partition had an independent rate multiplier with prior Γ(1,1), as well as stationary frequencies and exchangeability rates drawn from flat, symmetrical Dirichlet distributions. The branch-rate prior was Γ(7,7), normalized to a mean of 1 across all branches. Four independent runs were performed, which agreed. Nodes with posterior probability less than 0.95 were collapsed into polytomies.

CI assays

To test for cytoplasmic incompatibility (CI) in *D. seguyi* and *D. bocqueti*, we estimated the egg hatch frequencies from crosses between females without *Wolbachia* and males with *Wolbachia* (denoted CI) and the reciprocal compatible cross (CC) between females with and males without *Wolbachia*. With CI, we expect lower egg hatch from CI crosses than from CC crosses. To generate lines of both species without *Wolbachia*, we exposed *Wolbachia*-carrying lines to tetracycline-supplemented (0.03%) cornmeal medium (see Shropshire et al. 2021 (*24*) for details) for three generations. We confirmed the absence of *Wolbachia* in the treated flies using PCR within two generations of tetracycline treatment using primers for the *Wolbachia*-specific *wsp* gene (*25*, *26*) and a second reaction for the arthropod-specific 28S rDNA as a host control (*27*). Our PCR thermal profile began with 3 min at 94C, followed by 34 rounds of 30 sec at 94C, 30 sec at 55C, and 1 min and 15 sec at 72C. The profile finished with one round of 8 min at 72C. We visualized PCR products using 1% agarose gels that included a molecular weight ladder. The flies were maintained and experiments were conducted in an incubator at 25°C.

We reciprocally crossed *Wolbachia*-carrying *D. seguyi* and *D. bocqueti* lines to their tetracycline-treated conspecifics. Virgins were collected from each line and placed into holding vials for 48 hr. We set up each CI and CC cross with one female and one male in a vial containing a small spoon with cornmeal medium and yeast paste for 24 hr. Males and females were two days old at the beginning of these experiments. Each pair was transferred to a fresh vial every 24 hr for 5 days. We counted the number of eggs laid at the

time that adults were transferred to new vials and the number of eggs that hatched were scored after an additional 24 hrs. The data analyzed were hatch proportions for crosses across the 5-day period. To control for cases where females may not have been inseminated, we excluded crosses that produced fewer than 10 eggs. We used one-sided Wilcoxon tests to determine whether CI crosses produce lower egg hatch proportions than do CC crosses.

Extracting *cif* sequences

BLAST was used to identify contigs with *cif* sequences. The following *cifA* and *cifB* loci were used as query sequences: *w*Mel[T1], *w*Ri[T2], *w*No[T3], *w*Pip[T4], *w*Stri[T5], *w*Tri[T5], and *w*Bor[T5]. Geneious Prime(*28*) identified open reading frames (ORFs) with blast homology to *cif* sequences were extracted for downstream analyses. $cifB_{wMal[T1]}$ was the only *cif* gene we extracted that did not have a clear ORF associated with it, but there was sequence homology in the region. We assigned the *cif* sequences to Types (T1–T5) based on similarity to reference genes of each Type. The sources of *Wolbachia* genomes used in *cif* sequence analyses, and the *cifs* associated with each *Wolbachia* genome, are outlined in Table S1.

Extracting serine recombinase genes

We used the large serine recombinase to categorize *Woviruses* as sr1WO, sr2WO, sr3WO or sr4WO(*29*). We used the WOCauB3, WOVitA1, WOMelB, and WOFol2 serine recombinases as queries in BLAST searches. If the *Wolbachia* assembly clearly assigned a serine recombinase sequence to a phage, we assigned the phage to sr1WO, sr2WO, sr3WO or sr4WO based on the similarity to the reference sequences.

Phylogenetic topological identity

We tested for discordance between phylogenetic trees estimated from different data, e.g., *cifA* versus serine recombinase sequences within the same *Woviruses* using the SH (*30*) and AU (*31*) tests, as implemented in IQ-Tree (*32*). Unlike the topological-similarity tests described below, the null hypothesis in these tests is topological identity.

Phylogenetic topological similarity

We used normalized Clustering Information (CID), Jaccard-Robinson-Foulds (JRF), and Robinson-Foulds (RF) distances to test for similarity between pairs of trees, as described by Smith (2020) (*33*). All metrics were calculated using the TreeDist package in R (*34*). To normalize the distance metrics, we divided the observed value by the mean distance obtained from comparing 10,000 random tree pairs with equal numbers of leaves. The random trees were generated using the ape R package (*35*). We calculated *P*-values as the proportion of random trees of the same size as our data that produced distances below the observed distance. This tests the hypothesis that two trees are more similar than expected by chance.

Characterizing Cif protein structures

HHPred was run on a Linux kernel to identify putative functional domains using the Pfam-A_v35 and SCOPe70_2.07 databases (*36*). Only annotations with > 80% probability were considered. If alternative annotations produced $P > 0.8$, the annotation with the highest P was selected. AlphaFold2 (*37*) was used to predict Cif structures. Entries in the "reduced database" provided with AlphaFold prior to 5/10/22 were used to generate multiple sequence alignments (MSA) within AlphaFold. Five structures were generated for each protein and amber relaxation was performed to prevent unrealistic folding patterns. The five relaxed models were sorted by mean pLDDT and the top result is reported and used in other analyses. Protein structures were visualized using PyMol 2.5.2 (*38*). Cealign was used to align proteins relative to $Cif_{wMel[T1]}$ for imaging.

In PyMol, TM-scores were generated for pairwise-comparisons of Cif proteins to determine Cif structural similarities using the psico module (*39*). Each analysis was performed twice, switching which was the reference and target. This impacts the TM-score because the score is normalized to the length of the target protein. A Mann-Whitney-U test in R was used to compare the TM-scores from CifA and CifB comparisons – truncated proteins and proteins at the edge of a contig were removed from this analysis. The relationship between TM-score and percent identity was assessed using a Spearman correlation.

Characterizing Cif selective pressures.

To identify evidence of selection along the Cif proteins, we calculated the ratio of the number of non-synonymous substitutions per non-synonymous site (d_N) to the number of synonymous substitutions per synonymous site (d_S) using the Sliding Window Analysis of Ka and Ks (SWAKK) webserver (*40*). SWAKK calculates d_N/d_S by generating an alignment of two nucleotide sequences, mapping the alignment onto the tertiary structure, and calculating d_N/d_S with a 10 Å spherical sliding window across the reference structure. $cif_{wMel[T1]}$, $cif_{wRi[T2]}$, $cif_{wApo[T3]}$, $cif_{wTei[T4]}$, and $cifA_{wTri[T5]}$ were used as references for each *cif* Type. AlphaFold structures were used for tertiary mapping. All statistical analyses were performed using the median d_N/d_S for each site across pairwise comparisons. BCa 95% confidence intervals for d_N/d_S values were calculated using the boot package in R (*41*).

Figure generation

Figures were produced and/or edited in R, Figtree, Inkscape 1.1, Adobe Illustrator, and Keynote.

Supplementary Discussion

Recombination

We detected little evidence for recombination among our 20 *w*Mel-like *Wolbachia* presented in Fig. 1A (GARD: 0/411 genes, PHI test: 0/411 = 0%, Max χ^2 test: 3/411 = 0.7%, Neighbour Similarity Score: 0/411 = 0%). To test for recombination among more diverged variants, we completed a separate analysis that included *w*Mel and *w*Ri, three other group-A variants (*Wolbachia* associated with *Andrena hattorfiana*, *Anoplius nigerrimus*, and *Apoderus coryli*), and B-group *w*Mau (*16*, *17*). GARD found evidence of recombination in 728 genes, with recombination in more than half of these genes also supported by the tests implemented in PhiPack (PHI: 418/728, Max χ^2: 485/728, NSS: 520/728). Recombination was detected by all three tests in 353 genes. These 353 genes include 245 with a gene region where *w*Mel and *w*Ri are sister. Of these, 7 have a region where *w*Mel and *w*Mau are sister, 25 have a region where *w*Ri and *w*Mau are sister, 37 have a region where *w*Mel and *A. nigerrimus Wolbachia* are sister, and 38 have a region where *w*Ri and *A. nigerrimus Wolbachia* are sister. This generalizes prior results that used only a few genes and found support for pervasive recombination between relatively diverged *Wolbachia* (*26*, *42*, *43*), while confirming that our use criteria for selecting genes to produce phylograms indirectly selects for genes with little evidence for recombination.

Recombination between group-A and group-B *Wolbachia* could potentially influence estimated phylograms and chronograms. To test this hypothesis, we searched for genes with no evidence of recombination using the 15 *Wolbachia* analyzed in Meany et al. (2019) (*17*). Of 168 single-copy and equal-length genes, we discarded 14 that were shorter than 300 bp. All three statistical tests found no evidence for recombination in 51 of the remaining 154 genes. We used these 51 genes to re-estimate a phylogram and absolute chronogram with RevBayes 1.1.1. Four independent runs of each were performed and all agreed. With the exception of the placement of *w*AlbB, our phylogram estimated using only the 51 genes with no evidence for recombination recapitulates the topology estimated by Meany et al. (2019)(*17*). Our estimate of A-B group divergence time using only these 51 genes (~7-38 MYA) also

overlaps with our previous relaxed-clock analyses with branch-rate priors Γ(7,7) (~8-46 MYA) and Γ(2,2) (~6-36 MYA), as well as with our strict-clock analysis (~12-64 MYA) (*17*). This confirms that our previously estimated phylograms and chronograms are insensitive to the inclusion of genes with evidence of recombination.

Inferences from chronograms

Inferences from these chronograms could be limited with only one available *Wolbachia* sequence from most of these hosts. For instance, we cannot determine which *Wolbachia* infection was acquired first, whether two sister tips on our *Wolbachia* chronograms correspond to one of those hosts acquiring its *Wolbachia* (by either introgression or non-sexual horizontal transmission) from the host of the sister *Wolbachia* variant – or whether one or both *Wolbachia* were recently acquired from an unsampled intermediate host. Turelli *et al*. (2018) (*17*) proposed that *w*Ri in *D. simulans* was acquired horizontally from *D. ananassae*, because multiple *Wolbachia* sequences were available from each species, and the *w*Ri sequences formed a clade nested within a paraphyletic cluster containing the *Wolbachia* from *D. ananassae*. As noted by Richardson et al. (2012) (*7*), divergence times for extant *Wolbachia* variants in a host species may be much shorter than the duration of its current *Wolbachia* variant, because selective sweeps and genetic drift will eliminate sequence variation that indicates the duration of associations.

Fig. S2. illustrates the limited inferences about *Wolbachia* divergence and acquisition possible from individual *Wolbachia* sequences from various hosts. We consider four hypothetical host species with horizontally acquired *Wolbachia*. Host 1 acquires its *Wolbachia*, denoted w_1, at time T_1, and we denote by T_i(j » i) the time at which host i acquires its *Wolbachia*, denoted w_i, from host j. If we postulate $T_1 > T_2$(1 » 2) > T_3(1 » 3) > T_4(2 » 4), the resulting chronogram is Fig. S2. The chronogram provides no clue that variant w_1 was acquired first or that w_2 was acquired from host 1. Thus, we can infer only that all but one of the variants shown in Figs. 1A were acquired in the last 51–263 KY and that a transfer between a dipteran and a hymenopteran host occurred within the last 39–187 KY, possibly mediated by intermediate hosts. Moreover, the chronogram Fig. 1A is consistent with many of these hosts, for which we do not have multiple *Wolbachia* sequences, acquiring their current *Wolbachia* very recently, followed by spatial spread as observed for *Wolbachia* in several hosts (*23*).

Correction of Turelli et al. (2018) concerning the acquisition of *w*Spc

The analyses presented in our text deal with only a single *Wolbachia* sequence from each host species. As noted in Fig. S2, single sequences do not allow us to determine the direction or temporal order of *Wolbachia* acquisitions. In contrast, Turelli et al. (2018) (*6*) had multiple *Wolbachia* sequences from several species. Specifically, the *w*Ri sequences from *D. simulans* formed a clade nested within a paraphyletic cluster of *w*Ana sequences from *D. ananassae*. Given that *D. ananassae* and *D. simulans* span the *D. melanogaster* species group, which diverged on the order of 47 MYA (see Fig. 1C), introgression is impossible. Hence we conjectured that horizontal non-sexual transmission of *Wolbachia* occurred between *D. ananassae* and *D. simulans*, with *D. ananassae* as a plausible source. Turelli et al. (2018, Fig. 1) (*6*) also analyzed eight *Wolbachia* sequences from *D. suzukii* (*w*Suz) and one *D. subpulchrella* (*w*Spc). Two copies of *w*Suz from Asia (the other *w*Suz samples came from North and South America) formed a clade with the single *w*Spc, sister to the other six *w*Suz sequences. We used these data to conjecture that *D. suzukii* was a plausible source of the *Wolbachia* in *D. subpulchrella*. However, the two putative Asian *w*Suz sequences, obtained from GenBank, were in fact derived from *D. subpulchrella* specimens (China-AA27 and Korea-AA7). This misidentification was first kindly pointed out to us by Mathieu Gaultier, then confirmed by Joanna Chiu. With this correction, the data in Fig. 1 of Turelli (2018) produce sister clades of six *w*Suz and three *w*Spc, providing no information on the direction of transfer, as illustrated in Fig. S2.

CI assays

We secured *D. seguyi* and *D. bocqueti* stocks carrying *w*Mel-like *Wolbachia* from the National *Drosophila* Species Stock Center. To test for CI, we first treated each of these stocks with tetracycline to generate stocks without *Wolbachia*. Conspecific crosses between females without *Wolbachia* and males with *Wolbachia* (CI) produce lower egg hatch than do conspecific compatible crosses (CC) between females and males without *Wolbachia* for both *w*Seg in *D. seguyi* (CI egg hatch = 0.34 ± 0.21, $N = 14$; CC egg hatch = 0.89 ± 0.11, $N = 18$; $P < 0.001$) and *w*Bocq (CI egg hatch = 0.18 ± 0.24, $N = 13$; CC egg hatch = 0.60 ± 0.30, $N = 17$; $P = 0.002$), indicative of CI. This confirms two new *w*Mel-like *Wolbachia* systems that cause relatively strong CI.

***cif* sequence and Cif structure**

We used up to three pieces of information—strain, type designation, and copy number—in a subscript behind the gene name to describe specific *cifs*. For example, *w*Zta *Wolbachia* encode two T1 *cifA* variants that we denote $cifA_{w\mathrm{Zta[T1\text{-}1]}}$ and $cifA_{w\mathrm{Zta[T1\text{-}2]}}$. Cif proteins are diverse (Fig. S3) are subject to putative pseudogenization with early in-frame stop codons (discussed below). Putatively intact CifA proteins range from 344 to 493 aa ($\mathrm{CifA}_{w\mathrm{Zta[T4]}}$–$\mathrm{CifA}_{w\mathrm{Dal[T1\text{-}2]}}$) and CifB proteins are between 546 to 4433 aa ($\mathrm{CifB}_{w\mathrm{San[T4]}}$–$\mathrm{CifB}_{w\mathrm{Sbr[T5]}}$) (Fig. S4A). The shortest $\mathrm{CifB}_{[\mathrm{T5}]}$ ($\mathrm{CifB}_{w\mathrm{Tri[T5]}}$ = 2833 aa) is 1304 aa larger than the largest CifB from other Types ($\mathrm{CifB}_{w\mathrm{Zta[T1\text{-}2]}}$ = 1529 aa). CifA homologs lack confident structural-homology-based domain annotations (Probability > 0.8, see Söding et al. 2005 for a description of HHpred homology detection and structure prediction) (Fig. S4A) (*44*, *45*). Conversely, all CifB homologs encode a PD-(D/E)XK nuclease (Nuc hereafter). $\mathrm{CifB}_{[\mathrm{T1}]}$ encode a Ulp1 deubiquitilase (Dub hereafter), and $\mathrm{CifB}_{[\mathrm{T5}]}$ uniquely encode OTUB2 deubiquitilase, R12E2.13-like mammalian-wide interspersed repeat, BurrH DNA-binding, Latrotoxin_C toxin, and tetratricopeptide repeat domains (Fig. S4A).

To investigate Cif protein sequence and structural similarity, we calculated pairwise amino-acid identity (ID) and template-modeling scores (TM) (*46*). TM-scores indicate the degree of similarity between two superimposed structures, with TM = 0 indicating no similarity, TM = 1 corresponding to complete congruence, and TM > 0.5 indicating highly similar (*47*). We found that putatively intact $\mathrm{CifA}_{[\mathrm{T1}]}$ proteins are significantly more similar than $\mathrm{CifB}_{[\mathrm{T1}]}$ from the same pairs ($N = 18$) in terms of both sequence (95% BCa confidence intervals: $\mathrm{ID}_{\mathrm{CifA[T1]}} = 0.78 – 0.86$, $\mathrm{ID}_{\mathrm{CifB}} = 0.69–0.79$; $P = 0.0002$; Fig S3a) and AlphaFold structure ($\mathrm{TM}_{\mathrm{CifA}} = 0.89 – 0.92$, $\mathrm{TM}_{\mathrm{CifB}} = 0.56–0.62$, $P < 10^{-10}$; Fig. 3B). We observed similar trends across Cif Types ($N = 28$ pairs; Fig. S3A) and among 12 $\mathrm{Cif}_{[\mathrm{T1}]}$ from *w*Mel variants and six from *w*Ri variants (Fig. S3B,C).

Phage turnover

Between one and four sr1WO–sr3WO haplotypes are in each *w*Mel-like and *w*Ri-like *Wolbachia*. Specifically, sr1WO is found in all *w*Ri-like *Wolbachia* except for *w*Aur and *w*Tri, and *Wovirus* sr2WO is found in all *w*Ri-like variants except *w*Tri, as well as the *w*Mel-like *w*Tris and the four-member *w*SYTZ clade. The most common phage haplotype is sr3WO, present in all *w*Mel-like and *w*Ri-like *Wolbachia*, with one to three copies per *Wolbachia* genome (Table S1). Aside from sr1WO, which exhibits perfect phylogenetic congruence with their *w*Ri-like *Wolbachia* (Fig 1c), sr2WO ($CID = 0.49$, $P = 0.0004$; $JRF = 0.50$, $P = 0.04$; $RF = 0.67$, $P = 0.0003$) and sr3WO ($CID = 0.73$, $P = 0.0$; $JRF = 0.72$, $P = 0.12$; $RF = 0.94$, $P = 0.0$) demonstrate imperfect but non-random congruence with their respective hosts (Fig S4a,b) (Smith et al. 2020). We report two sr2WO haplotypes: the first is in the *w*Mel-like *w*SYTZ clade (23–133 KYA) and in the divergent *w*Mel-like *w*Tris, but absent in *w*Tris's closest relative *w*Rec, suggesting that either *w*Tris recently acquired this phage or it was present in the common ancestor of *w*Tris and *w*Rec but lost from *w*Rec in the last 28 to 166 KYA (Fig S4a); the second sr2WO haplotype is shared by all of our *w*Ri-like *Wolbachia*. As for sr3WO, *w*Ri-like *Wolbachia* harbor only a single sr3WO haplotype that is in

all *w*Ri-like *Wolbachia*, absent in all *w*Mel-like strains, and recently duplicated in *w*Ri, *w*Ana, and *w*Suz (13–69 KYA) (Fig S5b,c). Multiple closely related sr3WO variants are present among closely related *w*Mel-like *Wolbachia*, including one shared among all 20 strains (50.6–263 KYA), one in a clade of six strains excluding *w*Bor (39–187 KYA), one in the *w*SYTZ group (23–133 KYA), and one in *w*Seg and *w*Mal (2.9–19 KYA) (Fig S5b,c). We also discovered that five *Wolbachia* harbor an sr3WO haplotype absent in their nearest sampled relatives: *w*Inc (diverged from its closest relative 36–179 KYA), *w*Bor (37–176 KYA), *w*Bocq (3.3–25 KYA), *w*Sbr (37–181 KYA), and *w*Dal (39–187 KYA). In summary, these patterns indicate that while *Woviruses* rapidly turnover, some associate with our *w*Mel-like variants on the order of 10^5 years.

cif turnover among phages

Cooper et al. (2019) (*48*) found that a single sr3WO *Wovirus* in *w*STY contain both Type 1 and Type IV *cif* operons, whereas closely related *w*Mel does not contain a Type IV *cif*. This discovery has been confirmed with more complete *w*STY assemblies(*49*). Thus *cifs* clearly move in and out of *Woviruses*, precluding concordant *cis* and *Wovirus* phylogenies. We used our estimated phylogenies for sr3WO haplotypes (sr3 alleles) and $cifA_{[T1]}$ alleles from Fig. 4 to assess how commonly *cifs* move among *Woviruses*. Horizontal *cif* movement among phages will produce discordance between the phylogenies estimated from sr3 alleles versus *cifs*. The SH and AU tests assess discordance by determining whether data from one set of markers is compatible with the phylogeny estimated from the other. As shown in Fig. 3, there is clear discordance in the topologies of $cifA_{[T1]}$ and sr3WO among the *w*Mel-like variants (e.g., look at the placements of (*w*Zta, *w*Ara) in panels 4a versus 4b). The SH and AU tests assign $P < 10^{-6}$ to the fit of the more resolved sr3WO data to the less resolved $cifA_{[T1]}$ tree in Fig. 3.

Origin and movement of _cif_ operons in _w_SYT

The primary mode of *cif* movement is difficult to ascertain without a comprehensive comparative analysis of fully contiguous genomes and associated plasmids. However due to *cifs'* imperfect association with phages, and their proximity to IS elements, *cif* horizontal transfer may plausibly occur via phage-mediated and phage-independent mechanisms. Cooper et al. (2019) (*48*) hypothesized that the Type IV *cifs* in *w*Yak (and more broadly, *w*SYT) could have entered a phage that already carried Type I *cifs* via homologous recombination with extrachromosomal DNA excised by IS elements. This proposal was disputed by Baião et al. (2021) (*49*). Using better assemblies, they proposed that phage probably delivered Type IV *cifs* into the ancestor of the *w*SYT clade and *w*Mel that were then subsequently deleted in *w*Mel. Their proposal is based on the phylogenetic relationship of *octomom* WD0513 homologs. Their hypothesis is implausible for several reasons that become obvious when assessing their assemblies.

First, the Type IV *cifs* in *w*SYT and divergent B-group *w*Pip are < 3% diverged, while the surrounding phage genes in *w*Yak and *w*Pip are ~15% diverged (see Cooper et al. 2019, Figure 5) (*48*). This points to a common donor for the Type IV *cifs* in *w*Yak and *w*Pip that is distinct from the phages that currently contain them. The highly diverged (~15%) phage sequences between *w*Yak and *w*Mel following the *cifAB* genes (Cooper et al. 2019, Figure 5) (*48*) clearly indicate that this transfer did not occur in the ancestor of *w*SYTM followed by deletion from *w*Mel, as proposed by Baião et al. (2021) (*49*). Baião et al. (2021) (*49*) note that the divergence of the WD0513 gene between *w*Mel and *w*SYT is "much higher than most other parts of the genomes" (~15%) but still conclude that this Octomom associated gene is orthologous to the copy in *w*SYT. This proposal is unreasonable, as most of the *w*Mel and *w*SYT genomes are <1% diverged. Given that the 15% divergence for WD0513 between *w*Mel and *w*SYT is comparable to the divergence for the phage-associated genes flanking the Type IV *cifs* in *w*SYT, it is most plausible that WD0513 was transferred with these phage genes and the Type IV *cifs* from an unknown donor. Incidentally, WD0513 is immediately flanked by a transposon (Baião et al. 2021, Figure 2A) (*49*) which, once again, makes it difficult to ascertain whether the mechanism providing extrachromosomal DNA for homologous recombination is phage-, transposon-, or

plasmid-mediated. Our finding of discordance between sr3 and associated *cifA*$_{[T1]}$ phylograms (Fig. 3) generalizes our initial discovery of phage-independent *cif* transfer (*48*).

References:

1. N. Joshi, J. Fass, Sickle: A sliding-window, adaptive, quality-based trimming tool for FastQ files, version 1.33; https://github.com/najoshi/sickle.
2. S. D. Jackman, B. P. Vandervalk, H. Mohamadi, J. Chu, S. Yeo, S. A. Hammond, G. Jahesh, H. Khan, L. Coombe, R. L. Warren, I. Birol, ABySS 2.0: resource-efficient assembly of large genomes using a Bloom filter. *Genome Res.* **27**, 768–777 (2017).
3. T. Seemann, Prokka: Rapid prokaryotic genome annotation. *Bioinformatics* **30**, 2068–2069 (2014).
4. K. Katoh, D. M. Standley, MAFFT multiple sequence alignment software version 7: improvements in performance and usability. *Mol. Biol. Evol.* **30**, 772–780 (2013).
5. S. Hohna, M. J. Landis, T. A. Heath, B. Boussau, N. Lartillot, B. R. Moore, J. P. Huelsenbeck, F. Ronquist, RevBayes: Bayesian phylogenetic inference using graphical models and an interactive model-specification language. *Syst. Biol.* **65**, 726–736 (2016).
6. M. Turelli, B. S. Cooper, K. M. Richardson, P. S. Ginsberg, B. Peckenpaugh, C. X. Antelope, K. J. Kim, M. R. May, A. Abrieux, D. A. Wilson, M. J. Bronski, B. R. Moore, J.-J. Gao, M. B. Eisen, J. C. Chiu, W. R. Conner, A. A. Hoffmann, Rapid global spread of *w*Ri-like *Wolbachia* across multiple *Drosophila*. *Curr. Biol.* **28**, 963 (2018).
7. M. F. Richardson, L. A. Weinert, J. J. Welch, R. S. Linheiro, M. M. Magwire, F. M. Jiggins, C. M. Bergman, Population genomics of the *Wolbachia* endosymbiont in *Drosophila melanogaster*. *PLOS Genet.* **8**, e1003129 (2012).
8. A. Holmes, J. E. Haber, “Physical monitoring of HO-induced homologous recombination” in *DNA Repair Protocols: Eukaryotic Systems*, D. S. Henderson, Ed. (Humana Press, Totowa, NJ, 1999; https://doi.org/10.1385/1-59259-675-4:403)*Methods in Molecular Biology™*, pp. 403–415.
9. B. Howell-Adams, H. S. Seifert, Molecular models accounting for the gene conversion reactions mediating gonococcal pilin antigenic variation. *Mol. Microbiol.* **37**, 1146–1158 (2000).
10. P. F. M. Meeus, K. A. Brayton, G. H. Palmer, A. F. Barbet, Conservation of a gene conversion mechanism in two distantly related paralogues of *Anaplasma marginale*. *Mol. Microbiol.* **47**, 633–643 (2003).
11. P. D. Dixit, T. Y. Pang, F. W. Studier, S. Maslov, Recombinant transfer in the basic genome of *Escherichia coli*. *Proc. Natl. Acad. Sci.* **112**, 9070–9075 (2015).
12. D. A. Haake, M. A. Suchard, M. M. Kelley, M. Dundoo, D. P. Alt, R. L. Zuerner, Molecular evolution and mosaicism of leptospiral outer membrane proteins involves horizontal DNA transfer. *J. Bacteriol.* **186**, 2818–2828 (2004).
13. L. Baldo, S. Bordenstein, J. J. Wernegreen, J. H. Werren, Widespread recombination throughout *Wolbachia* genomes. *Mol. Biol. Evol.* **23**, 437–449 (2006).
14. S. L. Kosakovsky Pond, D. Posada, M. B. Gravenor, C. H. Woelk, S. D. W. Frost, GARD: a genetic algorithm for recombination detection. *Bioinforma. Oxf. Engl.* **22**, 3096–3098 (2006).
15. T. C. Bruen, H. Philippe, D. Bryant, A simple and robust statistical test for detecting the presence of recombination. *Genetics* **172**, 2665–2681 (2006).
16. E. Vancaester, M. Blaxter, Phylogenomic analysis of *Wolbachia* genomes from the Darwin Tree of Life biodiversity genomics project. *PLOS Biol.* **21**, e3001972 (2023).
17. M. K. Meany, W. R. Conner, S. Richter, J. A. Bailey, M. Turelli, B. S. Cooper, Loss of cytoplasmic incompatibility and minimal fecundity effects explain relatively low *Wolbachia* frequencies in *Drosophila mauritiana*. *Evolution* **73**, 1278–1295 (2019).
18. B. Misof, S. Liu, K. Meusemann, R. S. Peters, A. Donath, C. Mayer, P. B. Frandsen, J. Ware, T. Flouri, R. G. Beutel, O. Niehuis, M. Petersen, F. Izquierdo-Carrasco, T. Wappler, J. Rust, A. J. Aberer, U. Aspoeck, H. Aspoeck, D. Bartel, A. Blanke, S. Berger, A. Boehm,

T. R. Buckley, B. Calcott, J. Chen, F. Friedrich, M. Fukui, M. Fujita, C. Greve, P. Grobe, S. Gu, Y. Huang, L. S. Jermiin, A. Y. Kawahara, L. Krogmann, M. Kubiak, R. Lanfear, H. Letsch, Y. Li, Z. Li, J. Li, H. Lu, R. Machida, Y. Mashimo, P. Kapli, D. D. McKenna, G. Meng, Y. Nakagaki, J. Luis Navarrete-Heredia, M. Ott, Y. Ou, G. Pass, L. Podsiadlowski, H. Pohl, B. M. von Reumont, K. Schuette, K. Sekiya, S. Shimizu, A. Slipinski, A. Stamatakis, W. Song, X. Su, N. U. Szucsich, M. Tan, X. Tan, M. Tang, J. Tang, G. Timelthaler, S. Tomizuka, M. Trautwein, X. Tong, T. Uchifune, M. G. Walzl, B. M. Wiegmann, J. Wilbrandt, B. Wipfler, T. K. F. Wong, Q. Wu, G. Wu, Y. Xie, S. Yang, Q. Yang, D. K. Yeates, K. Yoshizawa, Q. Zhang, R. Zhang, W. Zhang, Y. Zhang, J. Zhao, C. Zhou, L. Zhou, T. Ziesmann, S. Zou, Y. Li, X. Xu, Y. Zhang, H. Yang, J. Wang, J. Wang, K. M. Kjer, X. Zhou, Phylogenomics resolves the timing and pattern of insect evolution. *Science* **346**, 763–767 (2014).

19. K. P. Johnson, C. H. Dietrich, F. Friedrich, R. G. Beutel, B. Wipfler, R. S. Peters, J. M. Allen, M. Petersen, A. Donath, K. K. O. Walden, A. M. Kozlov, L. Podsiadlowski, C. Mayer, K. Meusemann, A. Vasilikopoulos, R. M. Waterhouse, S. L. Cameron, C. Weirauch, D. R. Swanson, D. M. Percy, N. B. Hardy, I. Terry, S. Liu, X. Zhou, B. Misof, H. M. Robertson, K. Yoshizawa, Phylogenomics and the evolution of hemipteroid insects. *Proc. Natl. Acad. Sci. U. S. A.* **115**, 12775–12780 (2018).
20. K. M. Bayless, M. D. Trautwein, K. Meusemann, S. Shin, M. Petersen, A. Donath, L. Podsiadlowski, C. Mayer, O. Niehuis, R. S. Peters, R. Meier, S. N. Kutty, S. Liu, X. Zhou, B. Misof, D. K. Yeates, B. M. Wiegmann, Beyond *Drosophila*: resolving the rapid radiation of Schizophoran flies with phylotranscriptomics. *BMC Biol.* **19**, 23 (2021).
21. A. Suvorov, B. Y. Kim, J. Wang, E. E. Armstrong, D. Peede, E. R. R. D'Agostino, D. K. Price, P. Waddell, M. Lang, V. Courtier-Orgogozo, J. R. David, D. Petrov, D. R. Matute, D. R. Schrider, A. A. Comeault, Widespread introgression across a phylogeny of 155 *Drosophila* genomes. *Curr. Biol. CB* **32**, 111-123.e5 (2022).
22. W. R. Conner, E. K. Delaney, M. J. Bronski, P. S. Ginsberg, T. B. Wheeler, K. M. Richardson, B. Peckenpaugh, K. J. Kim, M. Watada, A. A. Hoffmann, M. B. Eisen, A. Kopp, B. S. Cooper, M. Turelli, A phylogeny for the *Drosophila montium* species group: A model clade for comparative analyses. *Mol. Phylogenet. Evol.* **158**, 107061 (2021).
23. F. C. De Ré, L. J. Robe, G. L. Wallau, E. L. S. Loreto, Inferring the phylogenetic position of the *Drosophila flavopilosa* group: Incongruence within and between mitochondrial and nuclear multilocus datasets. *J. Zool. Syst. Evol. Res.* **55**, 208–221 (2017).
24. J. D. Shropshire, E. Hamant, B. S. Cooper, Male age and *Wolbachia* dynamics: investigating how fast and why bacterial densities and cytoplasmic incompatibility strengths vary. *mBio* **12**, e02998-21 (2021).
25. H. R. Braig, W. G. Zhou, S. L. Dobson, S. L. O'Neill, Cloning and characterization of a gene encoding the major surface protein of the bacterial endosymbiont *Wolbachia pipientis*. *J. Bacteriol.* **180**, 2373–2378 (1998).
26. L. Baldo, N. Lo, J. H. Werren, Mosaic nature of the *Wolbachia* surface protein. *J. Bacteriol.* **187**, 5406–5418 (2005).
27. C. C. Nice, Z. Gompert, M. L. Forister, J. A. Fordyce, An unseen foe in arthropod conservation efforts: The case of *Wolbachia* infections in the Karner blue butterfly. *Biol. Conserv.* **142**, 3137–3146 (2009).
28. M. Kearse, R. Moir, A. Wilson, S. Stones-Havas, M. Cheung, S. Sturrock, S. Buxton, A. Cooper, S. Markowitz, C. Duran, T. Thierer, B. Ashton, P. Meintjes, A. Drummond, Geneious Basic: An integrated and extendable desktop software platform for the organization and analysis of sequence data. *Bioinformatics* **28**, 1647–1649 (2012).
29. S. R. Bordenstein, S. R. Bordenstein, Widespread phages of endosymbionts: Phage WO genomics and the proposed taxonomic classification of Symbioviridae. *PLOS Genet.* **18**, e1010227 (2022).

30. H. Shimodaira, M. Hasegawa, Multiple comparisons of log-likelihoods with applications to phylogenetic inference. *Mol. Biol. Evol.* **16**, 1114 (1999).
31. H. Shimodaira, An approximately unbiased test of phylogenetic tree selection. *Syst. Biol.* **51**, 492–508 (2002).
32. B. Q. Minh, H. A. Schmidt, O. Chernomor, D. Schrempf, M. D. Woodhams, A. von Haeseler, R. Lanfear, IQ-TREE 2: new models and efficient methods for phylogenetic inference in the genomic era. *Mol. Biol. Evol.* **37**, 1530–1534 (2020).
33. M. R. Smith, Information theoretic generalized Robinson-Foulds metrics for comparing phylogenetic trees. *Bioinformatics* **36**, 5007–5013 (2020).
34. M. R. Smith, R. Jonker, Y. Yang, Y. Cao, TreeDist: calculate and map distances between phylogenetic trees, version 2.6.3 (2023); https://cran.r-project.org/web/packages/TreeDist/index.html.
35. E. Paradis, S. Blomberg, B. Bolker [aut, cph, J. Brown, S. Claramunt, J. Claude, H. S. Cuong, R. Desper, G. Didier, B. Durand, J. Dutheil, R. J. Ewing, O. Gascuel, T. Guillerme, C. Heibl, A. Ives, B. Jones, F. Krah [aut, cph, D. Lawson, V. Lefort, P. Legendre, J. Lemon, G. Louvel, E. Marcon [aut, cph, R. McCloskey, J. Nylander, R. Opgen-Rhein, A.-A. Popescu, M. Royer-Carenzi, K. Schliep, K. Strimmer, D. de Vienne, ape: analyses of phylogenetics and evolution, version 5.7-1 (2023); https://cran.r-project.org/web/packages/ape/index.html.
36. L. Zimmermann, A. Stephens, S.-Z. Nam, D. Rau, J. Kuebler, M. Lozajic, F. Gabler, J. Soeding, A. N. Lupas, V. Alva, A completely reimplemented MPI bioinformatics toolkit with a new HHpred server at its core. *J. Mol. Biol.* **430**, 2237–2243 (2018).
37. J. Jumper, R. Evans, A. Pritzel, T. Green, M. Figurnov, O. Ronneberger, K. Tunyasuvunakool, R. Bates, A. Žídek, A. Potapenko, A. Bridgland, C. Meyer, S. A. A. Kohl, A. J. Ballard, A. Cowie, B. Romera-Paredes, S. Nikolov, R. Jain, J. Adler, T. Back, S. Petersen, D. Reiman, E. Clancy, M. Zielinski, M. Steinegger, M. Pacholska, T. Berghammer, S. Bodenstein, D. Silver, O. Vinyals, A. W. Senior, K. Kavukcuoglu, P. Kohli, D. Hassabis, Highly accurate protein structure prediction with AlphaFold. *Nature* **596**, 583–589 (2021).
38. Schrödinger, LLC, The PyMOL molecular graphics system, version 1.8 (2015).
39. T. Holder, Pymol ScrIpt COllection (PSICO), (2023); https://github.com/speleo3/pymol-psico.
40. H. Liang, W. Zhou, L. F. Landweber, SWAKK: a web server for detecting positive selection in proteins using a sliding window substitution rate analysis. *Nucleic Acids Res.* **34**, W382–W384 (2006).
41. A. Canty, boot: Bootstrap Functions (Originally by Angelo Canty for S), version 1.3-28.1 (2022); https://cran.r-project.org/web/packages/boot/index.html.
42. F. M. Jiggins, J. H. G. von der Schulenburg, G. D. D. Hurst, M. E. N. Majerus, Recombination confounds interpretations of *Wolbachia* evolution. *Proc. R. Soc. Lond. B Biol. Sci.* **268**, 1423–1427 (2001).
43. F. M. Jiggins, G. D. D. Hurst, Z. H. Yang, Host-symbiont conflicts: Positive selection on an outer membrane protein of parasitic but not mutualistic Rickettsiaceae. *Mol. Biol. Evol.* **19**, 1341–1349 (2002).
44. A. R. I. Lindsey, D. W. Rice, S. R. Bordenstein, A. W. Brooks, S. R. Bordenstein, I. L. G. Newton, Evolutionary genetics of cytoplasmic incompatibility genes *cifA* and *cifB* in prophage WO of *Wolbachia*. *Genome Biol. Evol.* **10**, 434–451 (2018).
45. J. Martinez, L. Klasson, J. J. Welch, F. M. Jiggins, Life and death of selfish genes: Comparative genomics reveals the dynamic evolution of cytoplasmic incompatibility. *Mol. Biol. Evol.* **38**, 2–15 (2021).
46. Y. Zhang, J. Skolnick, Scoring function for automated assessment of protein structure template quality. *Proteins* **57**, 702–710 (2004).

47. J. Xu, Y. Zhang, How significant is a protein structure similarity with TM-score = 0.5? *Bioinforma. Oxf. Engl.* **26**, 889–895 (2010).
48. B. S. Cooper, D. Vanderpool, W. R. Conner, D. R. Matute, M. Turelli, *Wolbachia* acquisition by *Drosophila yakuba*-clade hosts and transfer of incompatibility loci between distantly related *Wolbachia*. *Genetics* **212**, 1399–1419 (2019).
49. G. C. Baião, J. Janice, M. Galinou, L. Klasson, Comparative genomics reveals factors associated with phenotypic expression of *Wolbachia*. *Genome Biol. Evol.* **13**, evab111 (2021).

Figure S1

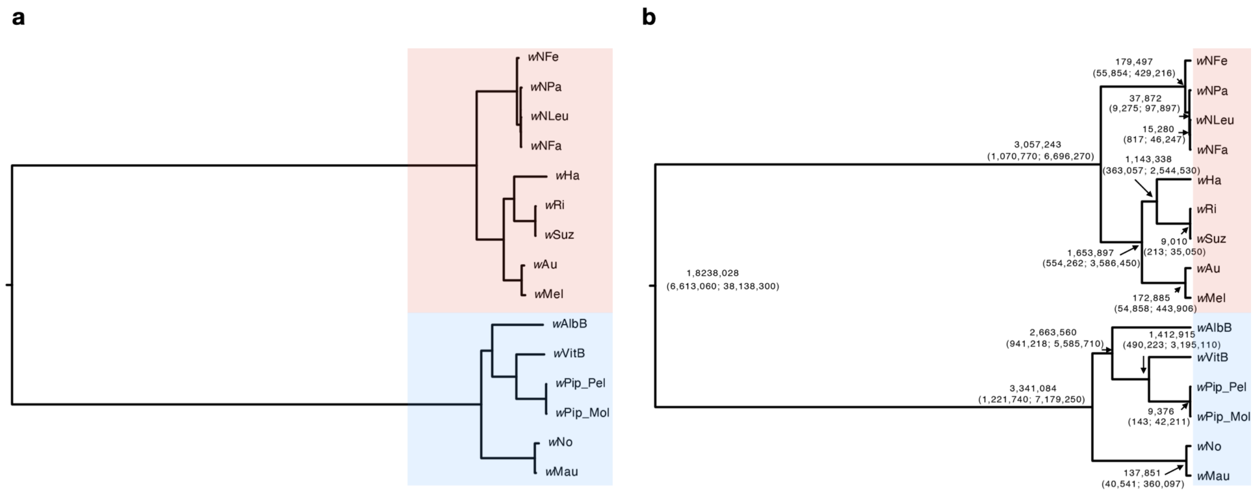

Fig. S1. Recombination does not influence estimated relationships or divergence times. **(A)** An estimated phylogram for group-A (red) and group-B (blue) *Wolbachia* strains using only the 51 genes with no evidence of recombination. Long branches separate the group-A and B clades, with significant variation in the substitution rates across branches. The topology here, with the exception of *w*AlbB placement, fully overlaps with the plylogram presented in Meany et al. (2019) that did not consider recombination. Meany et al. (2019) placed *w*AlbB outgroup to all other group-B *Wolbachia*. All nodes have Bayesian posterior of one, with the exception of the node distinguishing *w*Nfa and *w*NLeu (0.99) and the node distinguishing *w*AlbB from (*w*VitB,(*w*Pip_Pel, *w*Pip_Mol)) (0.98). Bayesian support less than 1 likely reflects real uncertainty in the placement of these strains. **(B)** An estimated chronogram that includes the same strains. Again, all nodes have Bayesian posterior of 1, with the exception of the node distinguishing *w*AlbB from (*w*VitB,(*w*Pip_Pel, *w*Pip_Mol)) (0.998) and the node distinguishing *w*Nfa and *w*NLeu (0.999). Point estimates for divergence times and their confidence intervals are presented for each node. Estimated divergence times broadly overlap with those estimated by Meany et al. (2019) without excluding genes with evidence for recombination.

Figure S2

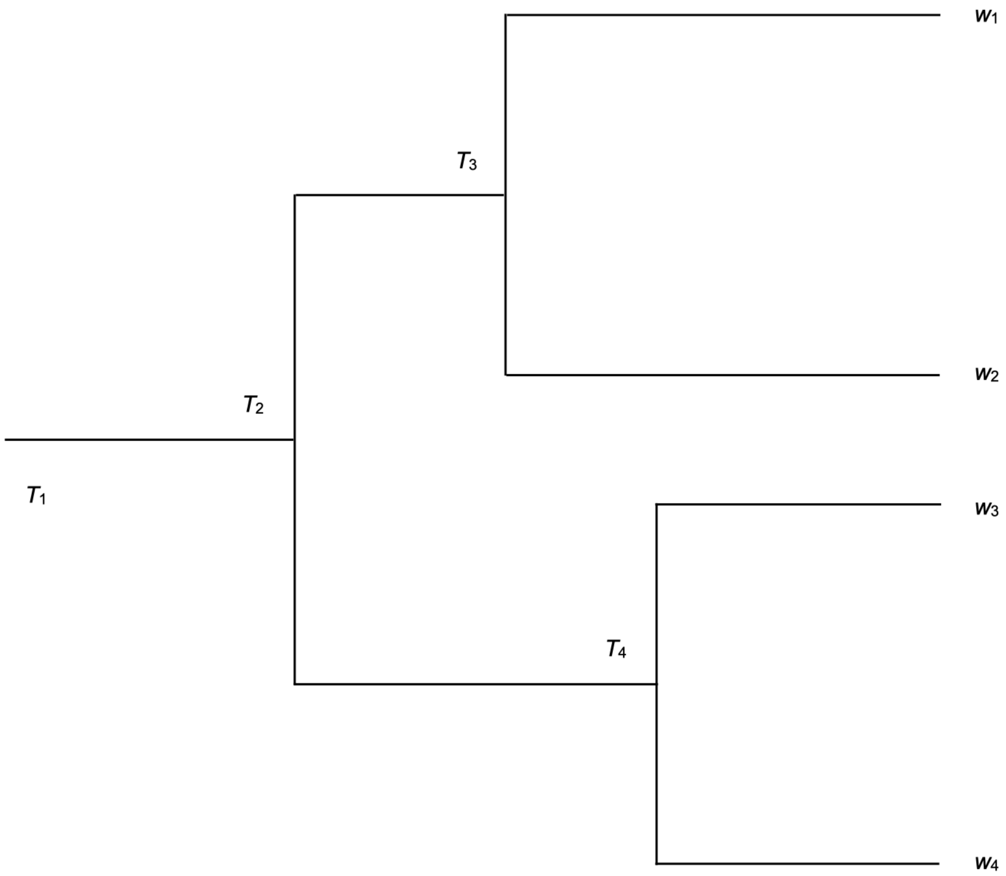

Fig. S2. An illustration of the limitations of inferences from the *Wolbachia* chronogram in Fig. 1a (See Supplementary Discussion).

Figure S3

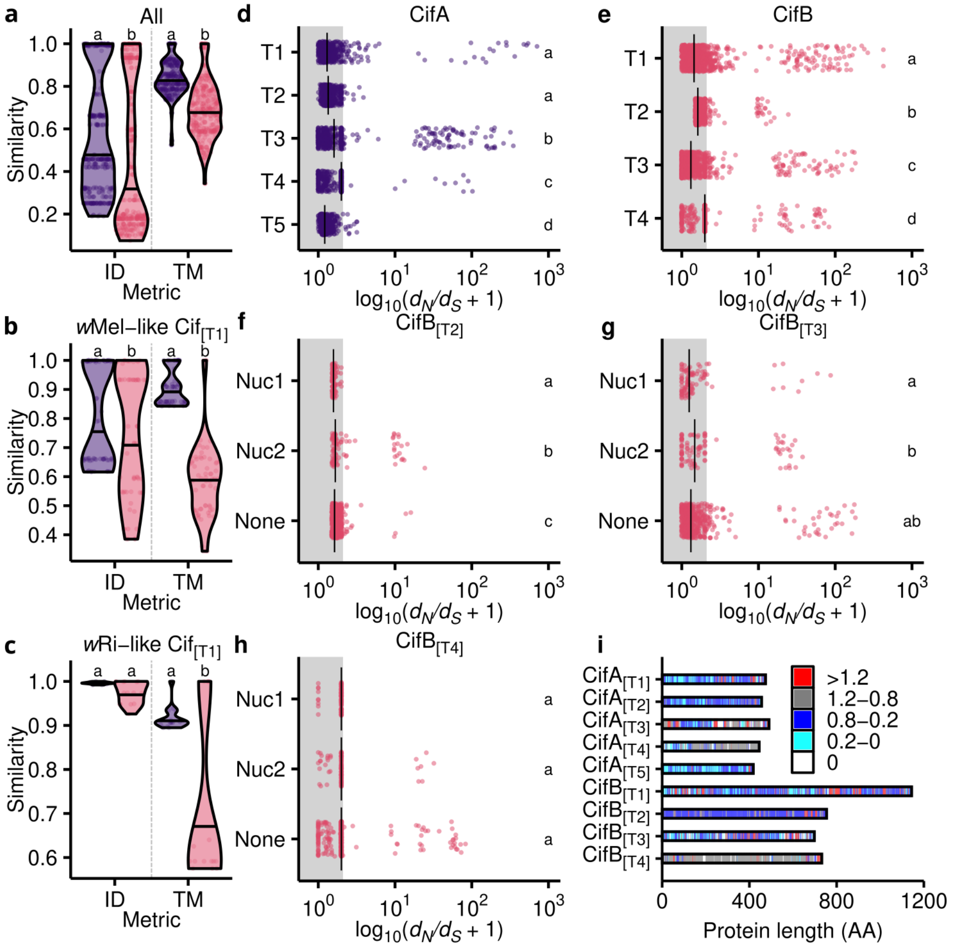

Fig. S3. Cif protein diversity and selective pressures. (**A-C**) The relationship between CifA (purple, left) and CifB (pink, right) pairwise sequence identity (ID) and structure similarity (TM). ID and TM are compared across (**a**) intact pairs excluding $\text{Cif}_{[\text{T5}]}$ ($N = 28$), (**B**) intact $\text{Cif}_{[\text{T1}]}$ pairs in *w*Mel-like *Wolbachia* ($N = 12$), and (**c**) intact $\text{Cif}_{[\text{T1}]}$ pairs in *w*Ri-like *Wolbachia* ($N = 6$). (**E-H**) Cif d_N/d_S using a 10 Å spherical sliding window in pairwise comparisons of $\text{Cif}_{w\text{Mel}[\text{T1}]}$, $\text{Cif}_{w\text{Ri}[\text{T2}]}$, $\text{Cif}_{w\text{Apo}[\text{T3}]}$, $\text{Cif}_{w\text{Teis}[\text{T4}]}$, and $\text{CifA}_{w\text{Mel}[\text{T5}]}$ to other T1 (CifA: $N = 9$; CifB: $N = 4$), T2 (CifA: $N = 5$; CifB: $N = 2$), T3 (CifA: $N = 5$; CifB: $N = 4$), T4 (CifA: $N = 5$; CifB: $N = 5$), and T5 (CifA: $N = 5$). $\text{CifB}_{[\text{T5}]}$ was excluded because full-length AlphaFold structures could not be constructed due to computational limitations. (**I**) Median d_N/d_S with a 10 Å window is displayed on linear protein schematics. Shared letters represent statistically similar groups determined by a Mann-Whitney U test (2 groups) or a Kruskal-Wallis and Dunn's multiple comparison test (>2 groups). *P*-values are in Table S3.

Figure S4

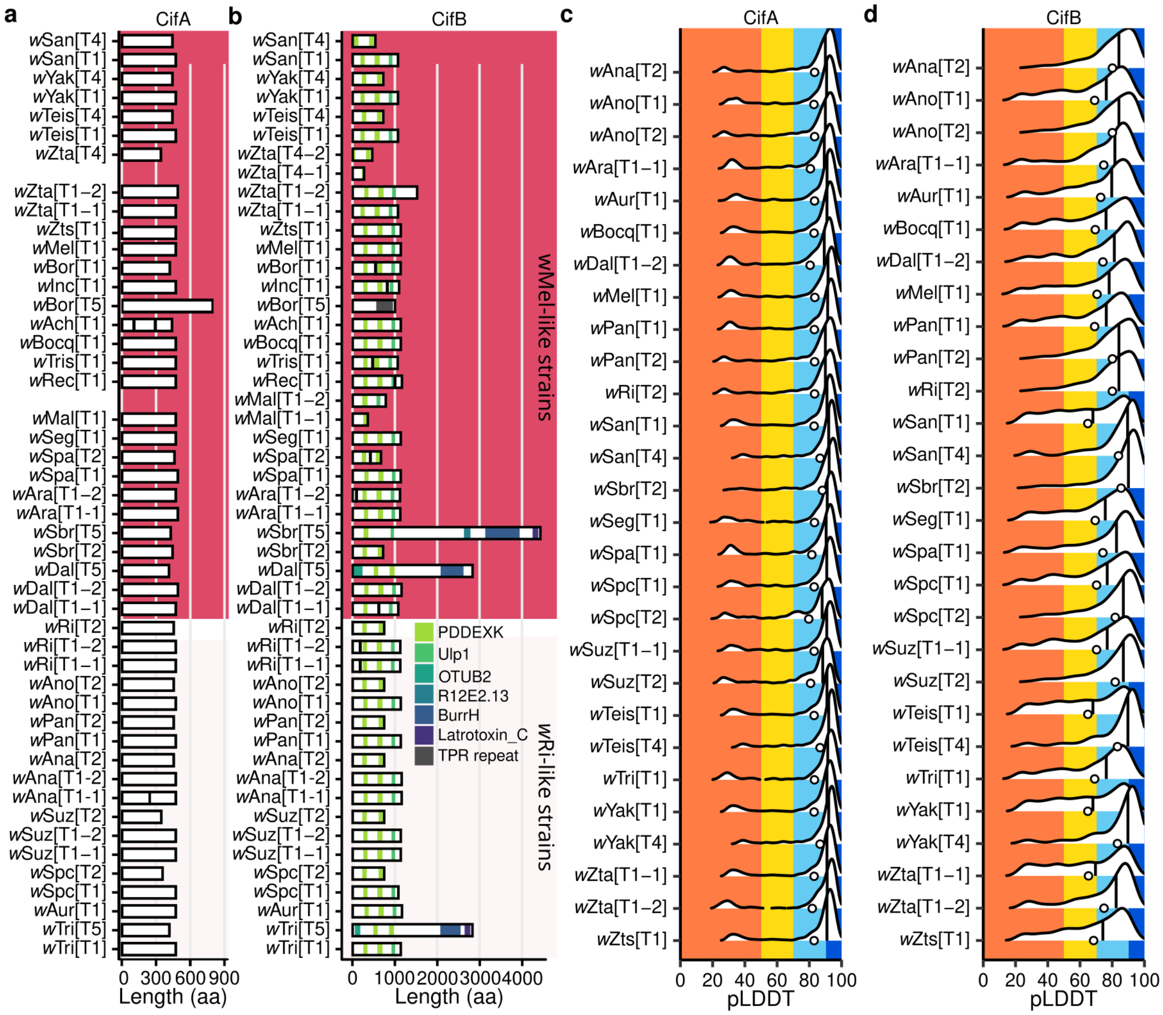

Fig. S4. Cif domain annotations and sequence/structure diversity. (**A**) CifA protein schematics. HHPred revealed no annotations with > 80% probability. (**B**) CifB protein schematics showing domains with > 80% probability. Relationship between Cif sequence and structure for Cif$_{[T1]}$ proteins of (**C**) *w*Mel-like and (**D**) *w*Ri-like *Wolbachia*. Structural confidence for (**C**) CifA and (**D**) CifB Alphafold structures. pLDDT is calculated per residue. pLDDT of 100 to 90 (dark blue), 90 to 70 (light blue), 70 to 50 (yellow), and 50 to 0 (orange) indicate very high, high, low, and very low confidence, respectively. Ridgeline plots illustrate the range and density of pLDDT values per protein. Vertical lines and dots represent median and mean pLDDT, respectively.

Figure S5

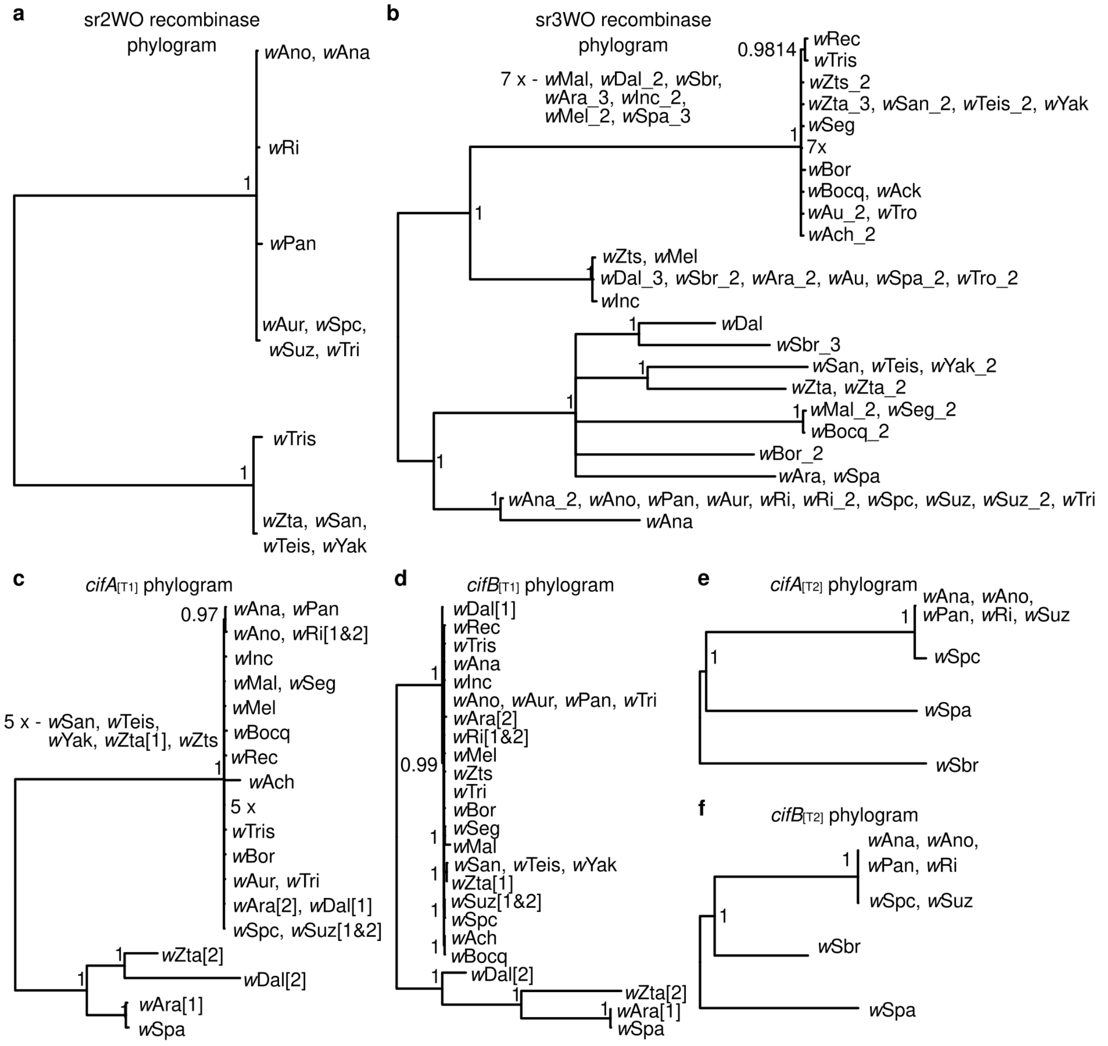

Fig. S5. Phage and *cif* phylograms. Serine recombinase phylogenies for homologs of the (**A**) sr2WO phage WOVitA and (**B**) sr3WO phage WOMelB. (**C**) $cifA_{[T1]}$, (**D**) $cifB_{[T1]}$, (**E**) $cifA_{[T2]}$, and (**F**) $cifB_{[T2]}$ phylograms with the relaxed clock GTR+G model. All phylograms are midpoint rooted. Node labels represent posterior probability. Identical sequences were collapsed into single tips and nodes with posterior probability < 0.95 were collapsed into polytomies.

Supplementary Legends

Table S1. *Wolbachia* strains, genomes, reproductive phenotypes, and *cifs*.

Table S2. Cif sequence and TM score similarity matrices. Pairwise percent identity of all (**A**) CifA and (**B**) CifB proteins reported in this study. Pairwise identity of (**C**) CifA and (**D**) CifB proteins included in the analysis of structure and sequence similarity. (**A-D**) Muscle5 was used to generate 100 multiple sequence alignments (MSA) and the alignment with the highest column confidence was used for downstream analyses. Pairwise identity was calculated as the percent of sites that are shared between pairs in the MSA relative to the length of the pair's alignment, including gaps. TM-scores from pairwise comparisons of (**E**) CifA and (**F**) CifB Alphafold structures.

Table S3. Complete information on phage and *cif* typing, and their association with each other and IS elements. **(A)** Summary of the CI phenotypes, *cif* composition, and phage composition for each *Wolbachia* strain in our study. **(B)** Summary of the serine recombinases, their closest *cif*s, and the genes adjacent to the *cifs* in either direction. **(C)** Summary of the *cifs* that we were unable to directly pair with a serine recombinase. We include the associated contig length, and the number of genes on that contig in either direction. **(D)** Summary of serine recombinases without a *cif* gene on the same contig, or where the serine recombinase is further from a *cif* gene than another serine recombinase in the genome. **(E)** Summary of *cifs* with nearby transposases and their identities. **(F)** Summary of the types of transposases found near *cifs* across our genomes, and their distances from focal *cifs*.

Table S4. Statistical results associated with main and supporting figures.

Movie S1. **d_N/d_S mapped on *w*Mel Cif AlphaFold structures.** Red, grey, blue, cyan, white, and black represent d_N/d_S values above 1.2, between 1.2 and 0.8, between 0.8 and 0.2, zero, and NA respectively. The N- and C- termini are displayed with sticks instead of spheres.
